# Supplementary material for: A nanounit strategy reverses immune suppression of exosomal PD-L1 and is associated with enhanced ferroptosis
Source: Nat Commun. 2021 Sep 30;12:5733. doi: 10.1038/s41467-021-25990-w (PMC8484261; doi:10.1038/s41467-021-25990-w)
Supplement: Supplementary file 1 — Supplementary infomation [file 41467_2021_25990_MOESM1_ESM.pdf]

Supplementary Information

A nanounit strategy reverses immune suppression of exosomal PD-L1 and is associated with enhanced ferroptosis

Guohao Wang[1,2,4], Lisi Xie[1,2,4], Bei Li [1,2,4], Wei Sang[1,2], Jie Yan[1,2], Jie Li[1,2], Hao Tian[1,2], Wenxi Li[1,2], Zhan Zhang[1,2], Ye Tian[1,2], and Yunlu Dai[1,2,3]*

[1]Cancer Centre, Faculty of Health Sciences, University of Macau, Macau SAR 999078, China.
[2]Institute of Translational Medicine, Faculty of Health Sciences, University of Macau, Macau SAR 999078, China.

[3]MoE Frontiers Science Center for Precision Oncology, University of Macau, Macau SAR 999078, China

[4]These authors contributed equally to this work.

* To whom correspondence should be addressed. Tel: (+) 853-8822 4881, Fax: (+) 853-8822 2314; E-mail: yldai@um.edu.mo (Y.D.)

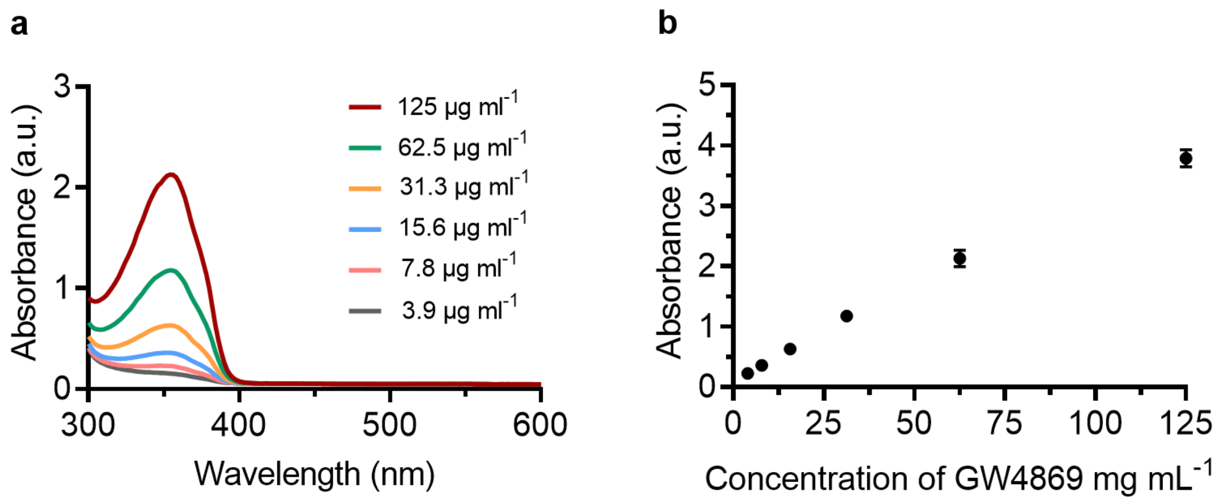

Supplementary Fig. 1. a The absorbance spectra of GW4869 at a series of concentrations. **b** Standard curve of GW4869 using UV spectrometry at 354 nm. Y=0.03X+0.13 (Y: UV value, X: concentration (μg ml^{-1}).

Table 1. GW4869 Loading efficiency in HGF

GW4869:HACA-Fe (w/w)	Loading content (%)	Loading efficiency (%)
1:9	7.56	75.60
1:4	16.35	81.75
2:3	22.36	55.9

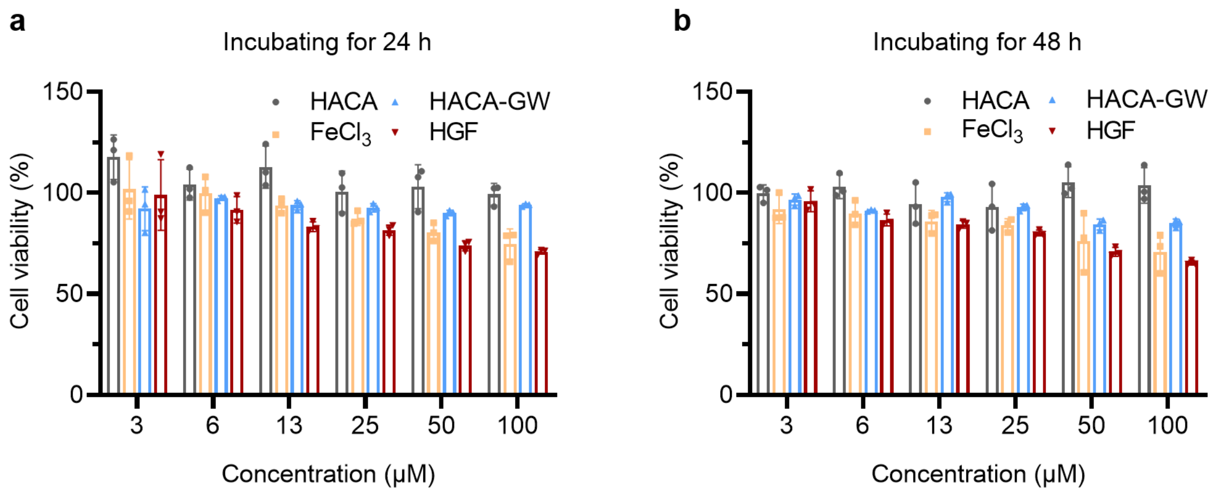

Supplementary Fig. 2. Cytotoxicity of HACA, $FeCl_3$, HACA-GW and HGF NPs on B16F10 cells by MTT assay after incubating for 24 h (**a**) and 48 h (**b**). n = 3 biologically independent cells per group. Data were presented as mean ± SD.

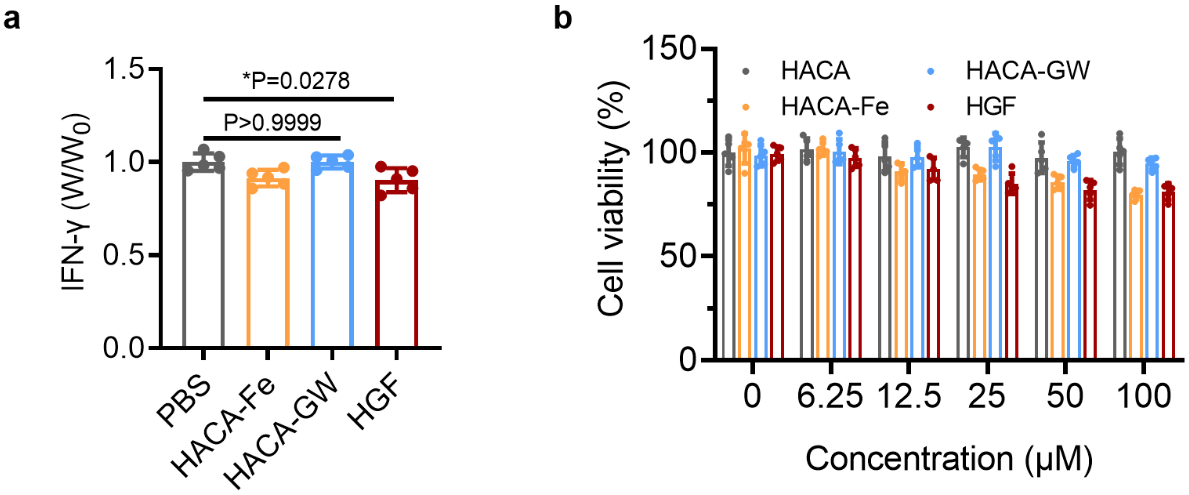

Supplementary Fig. 3. a Relative IFN-γ release from $CD8^+$ T cells incubated in PBS, HACA-Fe, HACA-GW and HGF. **b** Viability of $CD8^+$ T cells after incubating with HACA, HACA-Fe, HACA-GW and HGF NPs at diverse concentrations for 48 h. n=5. Data was presented as mean ± SD. The analysis method was One-way ANOVA with Tukey's post hoc test. Significance was presented as *P < 0.05, **P < 0.01, ***P < 0.001 and ****P < 0.0001.

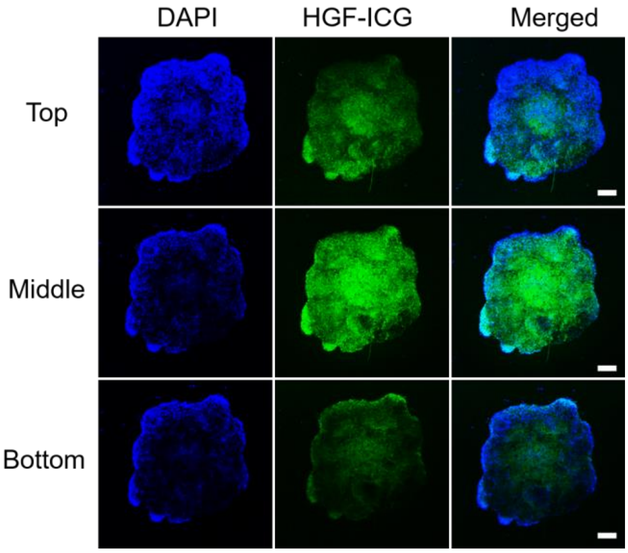

Supplementary Fig. 4. Representative confocal microscopy images of a 3D tumoral spheroid from various layers (top, middle, and bottom). The scale bar: 100 μm. Images were representative of three experiments.

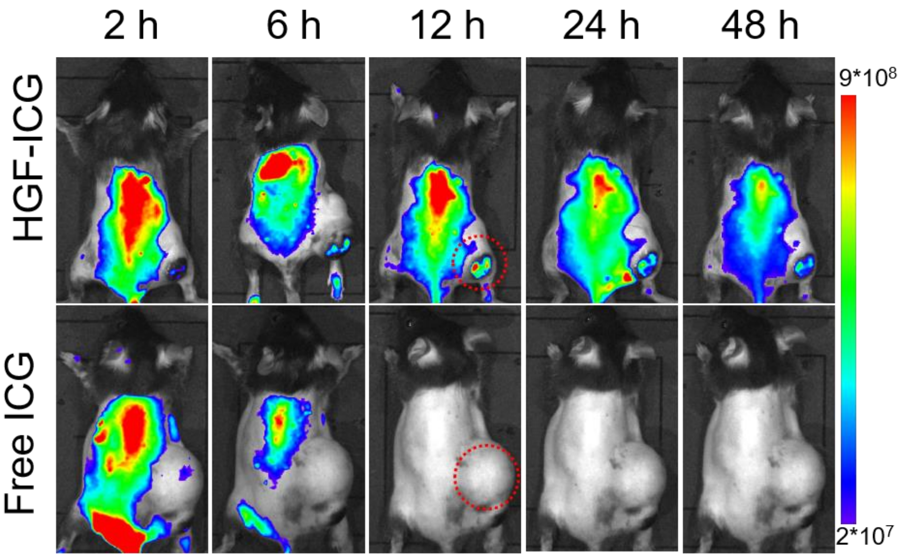

Supplementary Fig. 5 (related to Fig. 2). The different timepoints' in vivo fluorescent images of B16F10 tumor-bearing mice after injection with free ICG and ICG labeled HGF NPs. The red circle indicated the location of the tumor. Images are representative of three biologically independent animals from each group.

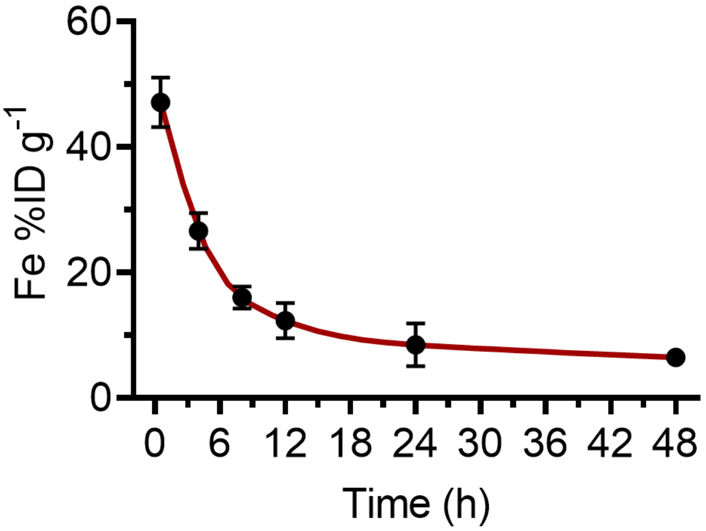

Supplementary Fig. 6 (related to Fig. 2). Blood circulation curve of HGF NPs in mice through measuring the blood concentration of Fe at 0.5, 4, 8, 12, 24 and 48 h. Data were presented as mean values ± SD (n=4).

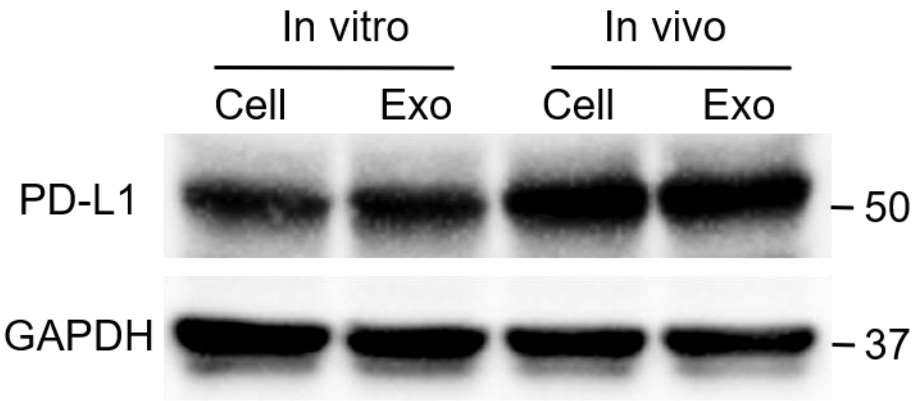

Supplementary Fig. 7 (related to Fig. 3). Immunoblots for PD-L1 in the whole cell lysate and purified exosomes from mouse melanoma B16F10 cells and tissue. The same amount of protein was loaded in each lane. Images were representative of three experiments.

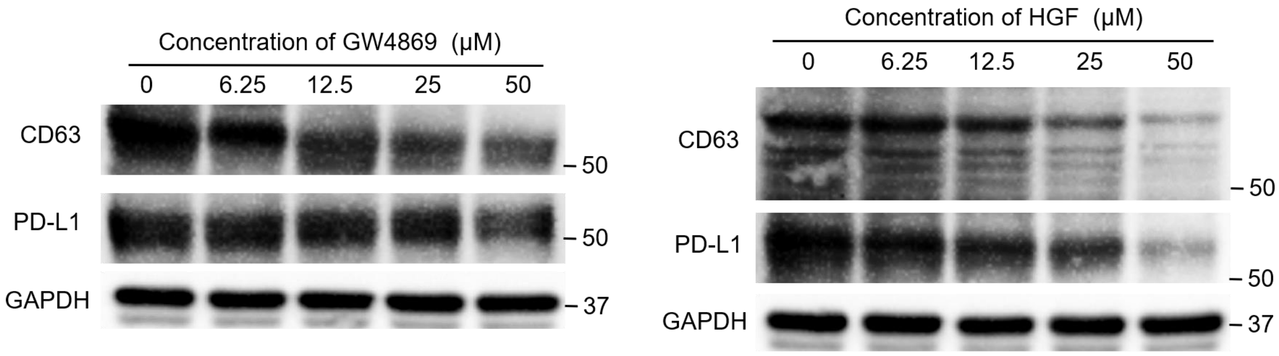

Supplementary Fig. 8 (related to Fig. 3). Western blot analysis for exosome marker CD63 and PD-L1 in the medium of B16F10 cells after treatment at different concentrations of HGF and GW4869. GAPDH served as a control. Images were representative of three experiments.

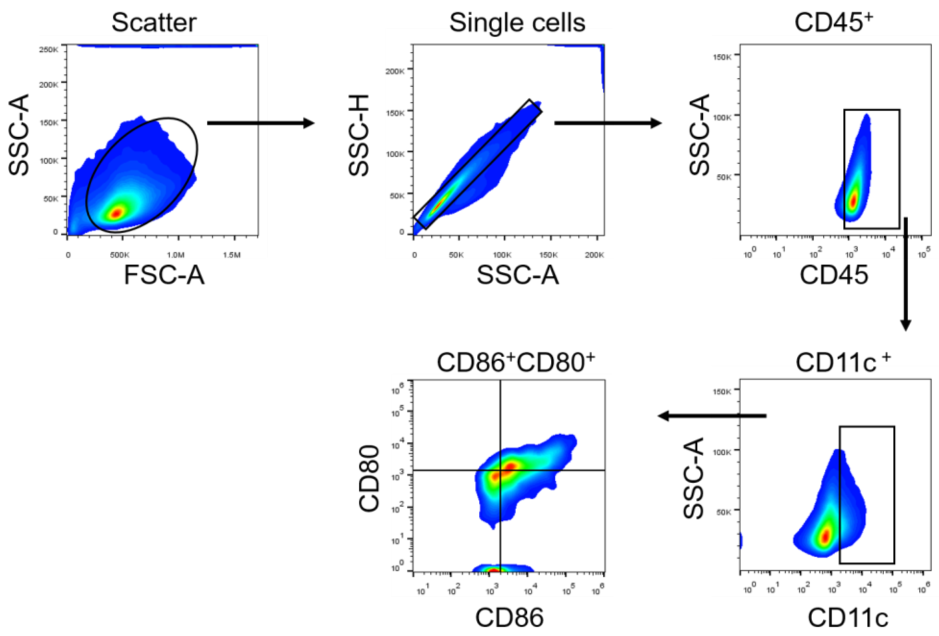

Supplementary Fig. 9 (related to Fig. 3). Representative flow cytometry gating strategies for CD11c$^+$CD80$^+$CD86$^+$ activated DCs panel.

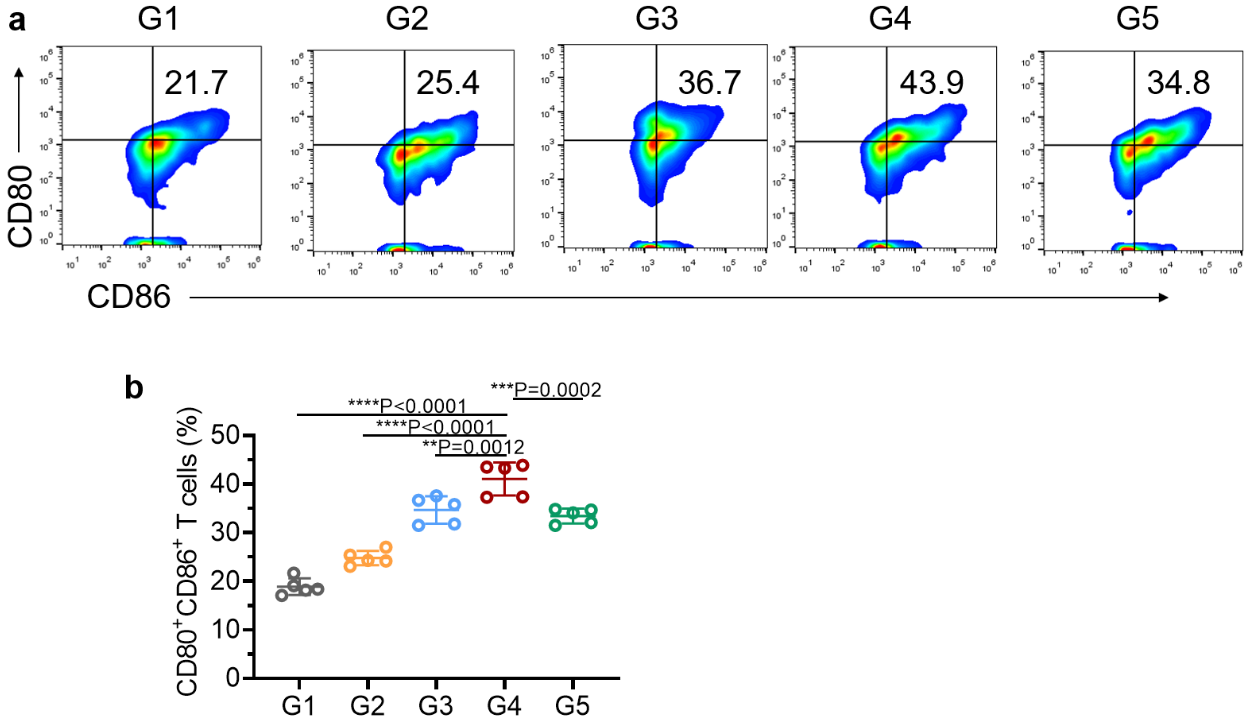

Supplementary Fig. 10 (related to Fig. 3). Representative flow cytometric plots (**a**) and the quantification (**b**) of matured DCs in TDLN after treatment. G1, PBS; G2, HACA-Fe; G3, HACA-GW; G4, HGF; G5, HGF+liproxstatin. n = 5 biologically independent animals per group. Data were presented as mean ± SD. Statistically significant differences between groups were identified by one-way ANOVA with Tukey's post hoc test. *P < 0.05, **P < 0.01, ***P < 0.001 and ****P < 0.0001.

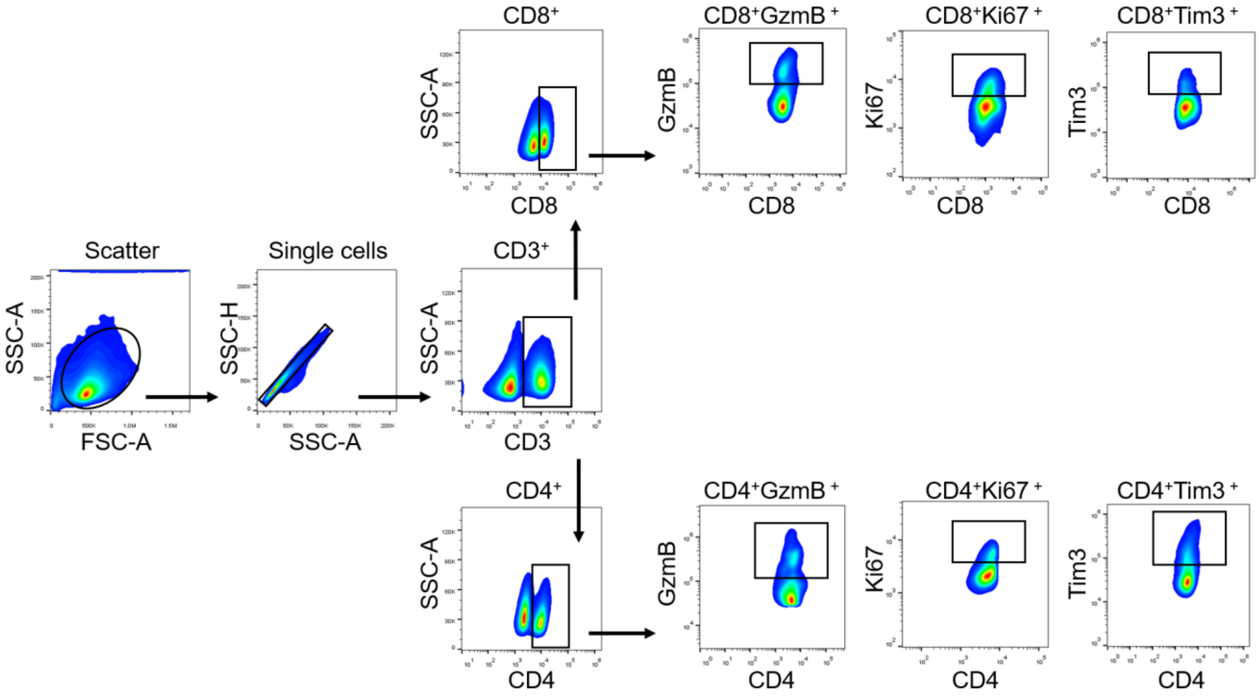

Supplementary Fig. 11 (related to Fig. 3). Representative flow cytometry gating strategies for $CD3^{+}CD4^{+}GzmB^{+}$, $CD3^{+}CD4^{+}Ki67^{+}$, $CD3^{+}CD4^{+}Tim3^{+}$, $CD3^{+}CD8^{+}GzmB^{+}$, $CD3^{+}CD8^{+}Ki67^{+}$ and $CD3^{+}CD8^{+}Tim3^{+}$ T cells panel in TDLN.

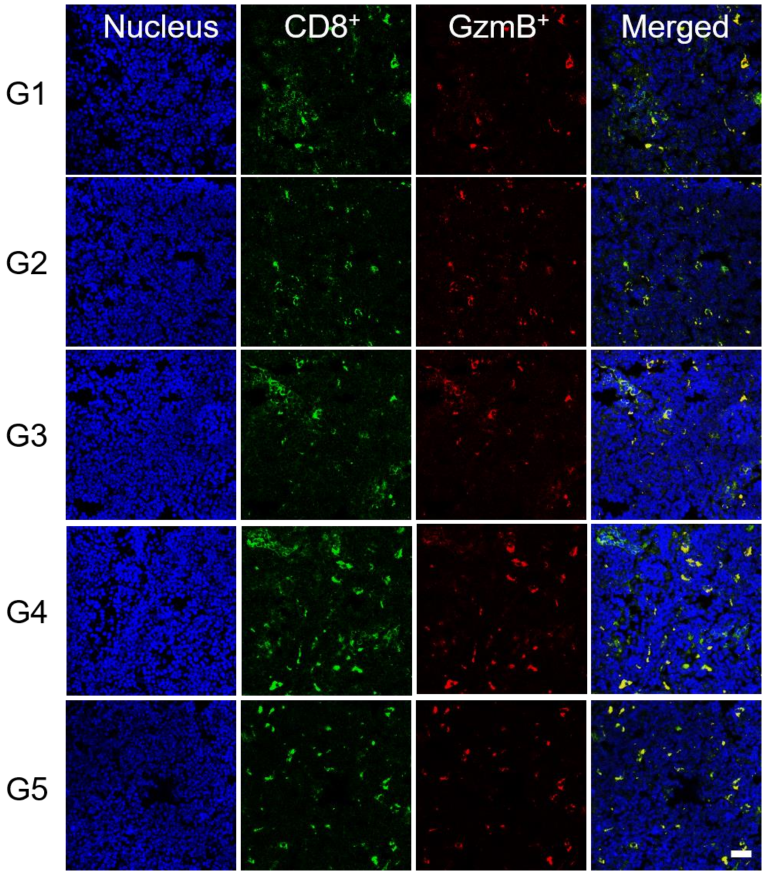

Supplementary Fig. 12 (related to Fig. 3). Representative CLSM images of $CD8^+GzmB^+$ T cells in TDLN after staining with DAPI (blue), $CD8^+$ antibody (green) and $GzmB^+$ antibody (red) respectively. G1, PBS; G2, HACA-Fe; G3, HACA-GW; G4, HGF; G5, HGF+liproxstatin. The scale bar: 50 μm. n = 3 biologically independent animals per group.

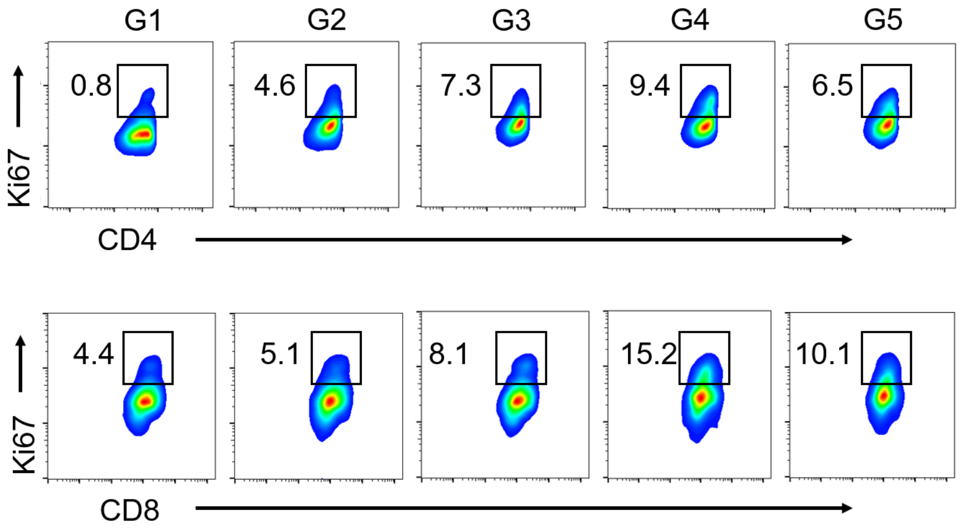

Supplementary Fig. 13 (related to Fig. 3). Representative flow cytometry plots of $CD3^+CD8^+Ki67^+$ and $CD3^+CD4^+Ki67^+$ T cells in TDLN. In flow cytometry plots, percentage of cells in the adjacent bound area was indicated. G1, PBS; G2, HACA-Fe; G3, HACA-GW; G4, HGF; G5, HGF+liproxstatin.

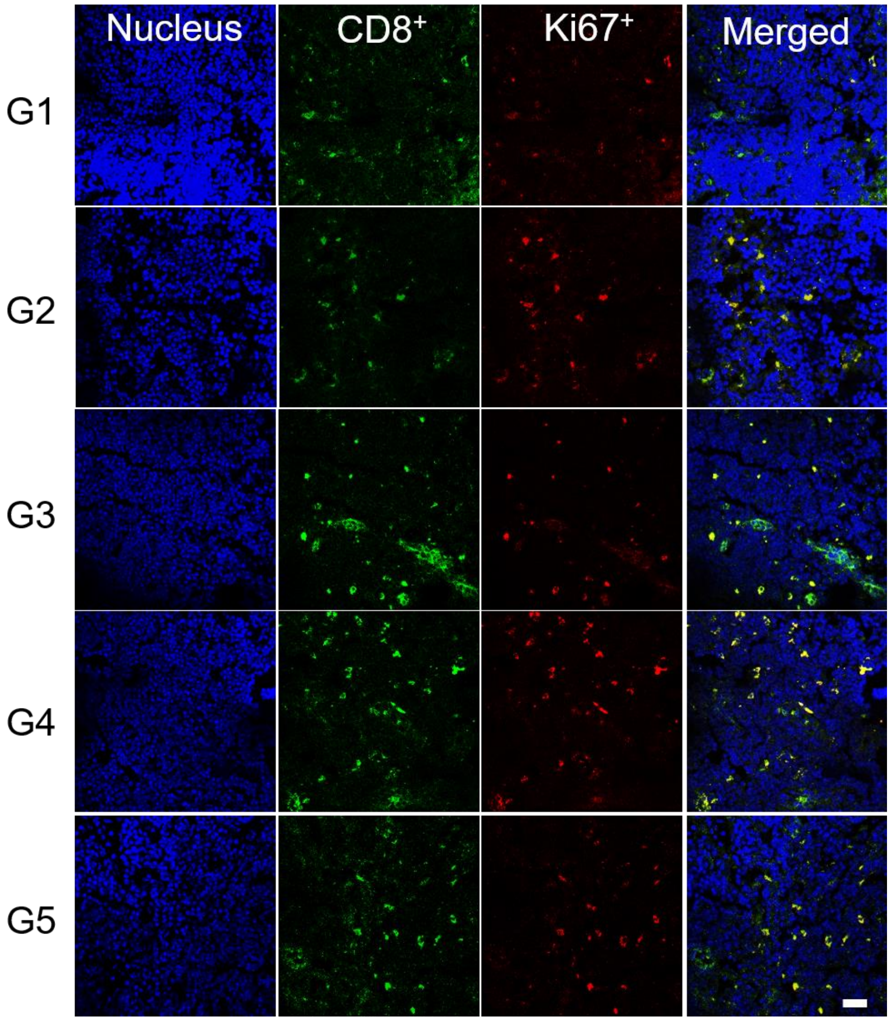

Supplementary Fig. 14 (related to Fig. 3). Representative CLSM images of $CD8^{+}Ki67^{+}$ T cells in TDLN after staining with DAPI (blue), $CD8^{+}$ antibody (green) and $Ki67^{+}$ antibody (red) respectively. G1, PBS; G2, HACA-Fe; G3, HACA-GW; G4, HGF; G5, HGF+liproxstatin. The scale bar: 50 μm. n = 3 biologically independent animals per group.

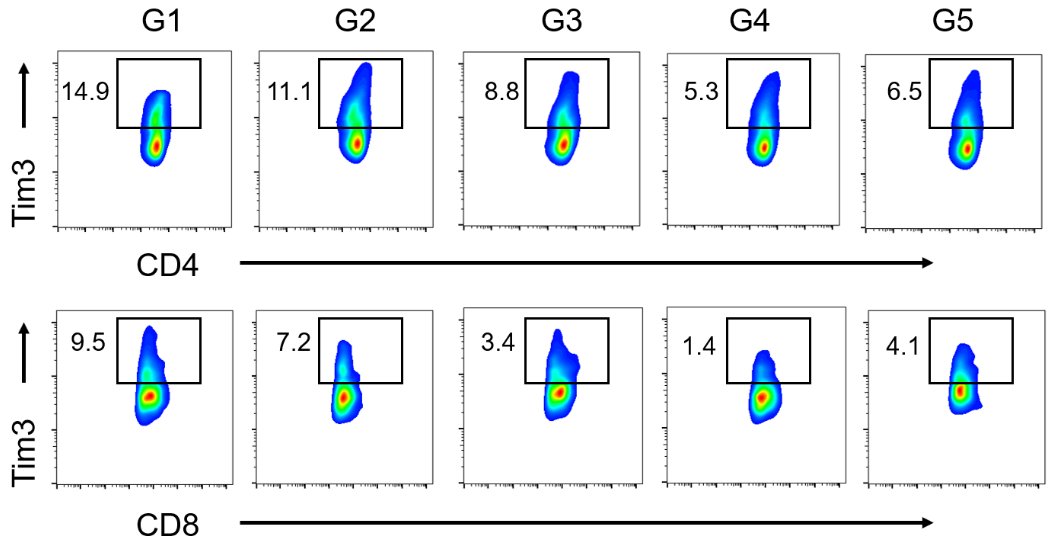

Supplementary Fig. 15 (related to Fig. 3). Representative flow cytometric plots of Tim3$^+$ cells in CD4$^+$ and CD8$^+$ T cells in TDLN. G1, PBS; G2, HACA-Fe; G3, HACA-GW; G4, HGF; G5, HGF+liproxstatin.

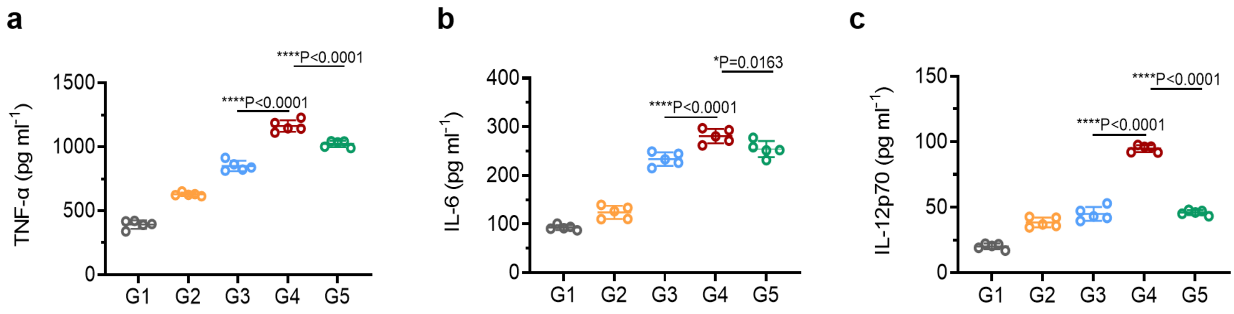

Supplementary Fig. 16 (related to Fig. 3). Serum cytokine concentrations of TNF-α (**a**), IL-6 (**b**) and IL-12p70 (**c**) at designated time points after treatment. G1, PBS; G2, HACA-Fe; G3, HACA-GW; G4, HGF; G5, HGF+liproxstatin. n = 5 biologically independent animals per group. Data were presented as mean ± SD. Statistically significant differences between groups were identified by one-way ANOVA with Tukey's post hoc test. *P < 0.05, **P < 0.01, ***P < 0.001 and ****P < 0.0001.

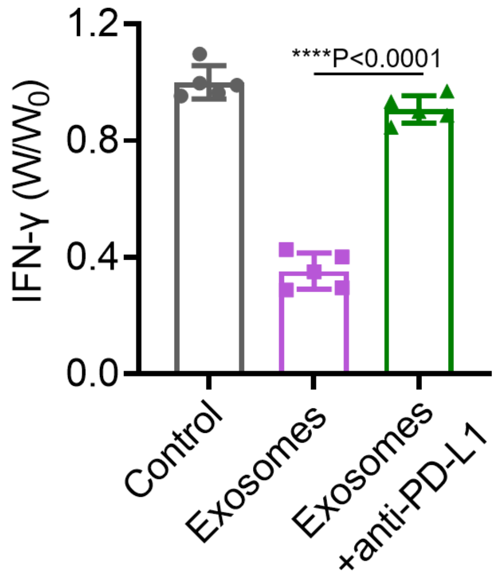

Supplementary Fig. 17 (related to Fig. 4). IFN-γ level released from CD8$^+$ T cells (stimulated with anti-CD3/CD28 antibodies) after treatment with PBS (Control), B16F10 cell-derived exosomes (Exosomes), B16F10 cell-derived exosomes with blocking PD-L1 antibodies (Exosomes+anti-PD-L1). n = 5 biologically independent samples per group. Data were presented as mean ± SD. Statistically significant differences between groups were identified by one-way ANOVA with Tukey's post hoc test. *P < 0.05, **P < 0.01, ***P < 0.001 and ****P < 0.0001.

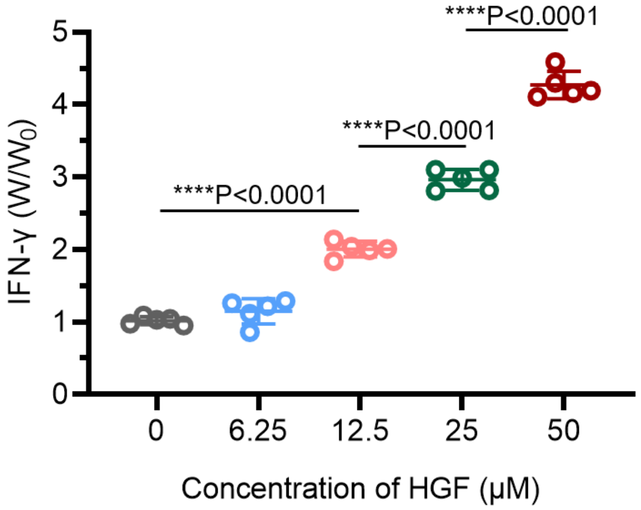

Supplementary Fig. 18 (related to Fig. 4). Relative IFN-γ released from CD8$^+$ T cells incubated with exosomes isolated from B16F10 cells of different conditions. n = 5 biologically independent samples per group. Data were presented as mean ± SD. Statistically significant differences between groups were identified by one-way ANOVA with Tukey's post hoc test. *P < 0.05, **P < 0.01, ***P < 0.001 and ****P < 0.0001.

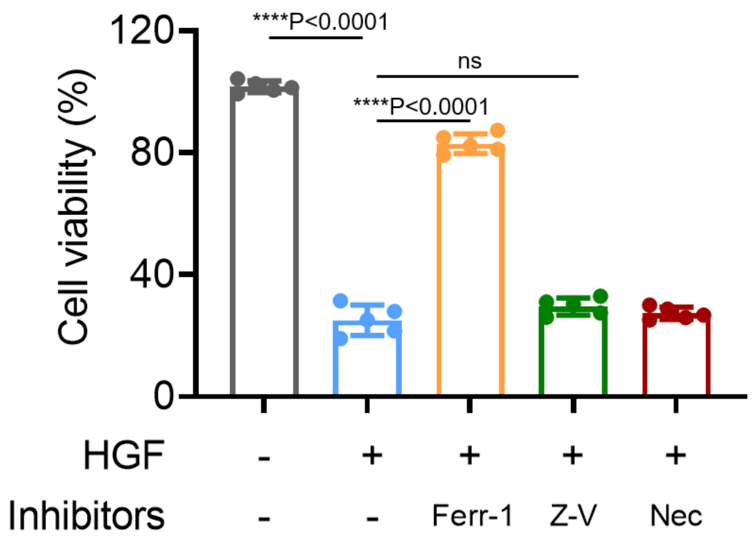

Supplementary Fig. 19 (related to Fig. 4). HGF NPs induced ferroptotic cell death in B16F10 cells. Cell viability measurements in B16F10 cells treated with HGF and cell death inhibitors for 24 h. Ferr-1, 1 μM ferrostatin-1 (inhibitor of ferroptosis); Z-V, 20 μM Z-VAD-FMK (inhibitor of apoptosis); Nec, 2 μM necrostatin-1s (inhibitor of necroptosis). n = 5 biologically independent samples per group. Data were presented as mean ± SD. Statistically significant differences between groups were identified by one-way ANOVA with Tukey's post hoc test. $*P < 0.05$, $**P < 0.01$, $***P < 0.001$ and $****P < 0.0001$.

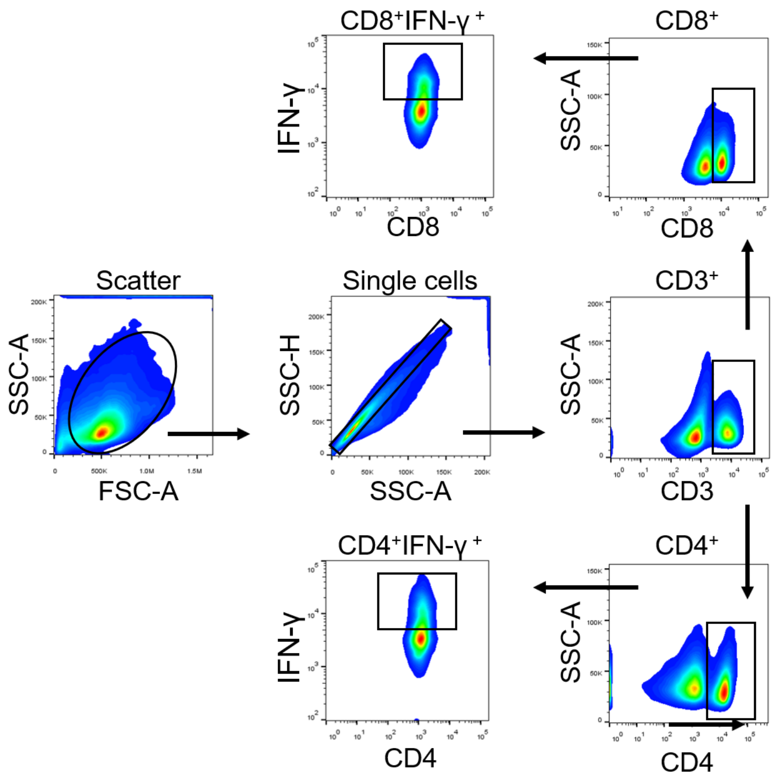

Supplementary Fig. 20 (related to Fig. 4). Representative flow cytometry gating strategies for CD4^{+}IFN-γ^{+} T cells and CD8^{+}IFN-γ^{+} T cells panel in TDLN.

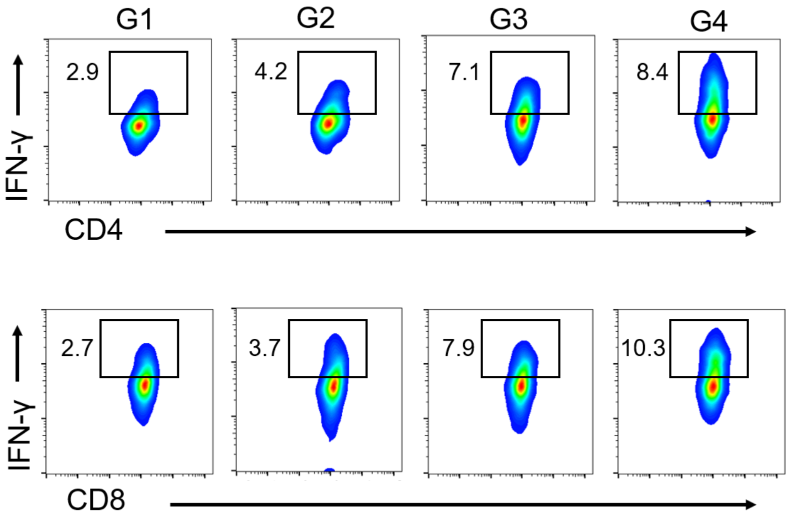

Supplementary Fig. 21 (related to Fig. 4). Representative flow cytometry plots of IFN-γ^{+} T cells after gating on CD3^{+}CD4^{+} T cells and IFN-γ+ T cells after gating on CD3^{+}CD8^{+} T cells in TDLN. G1, PBS; G2, HACA-Fe; G3, HACA-GW; G4, HGF.

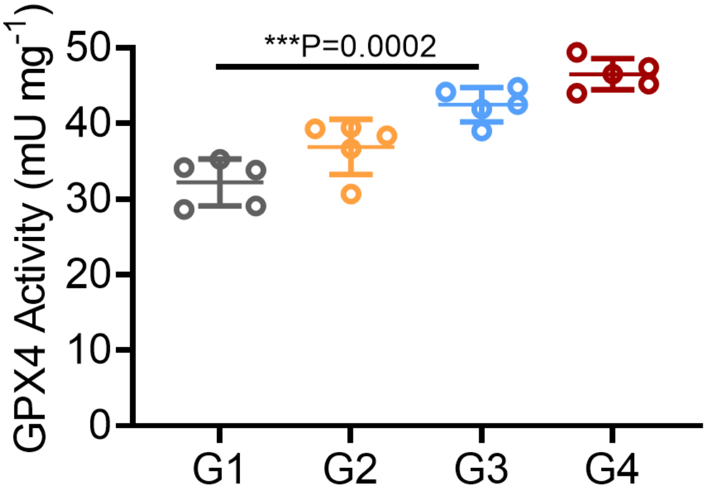

Supplementary Fig. 22 (related to Fig. 4). GPX4 activity in T cells collected from lymph node after treatment. G1, PBS; G2, HACA-Fe; G3, HACA-GW; G4, HGF. n = 5 biologically independent animals per group. Data were presented as mean ± SD. Statistically significant differences between groups were identified by one-way ANOVA with Tukey's post hoc test. *P < 0.05, **P < 0.01, ***P < 0.001 and ****P < 0.0001.

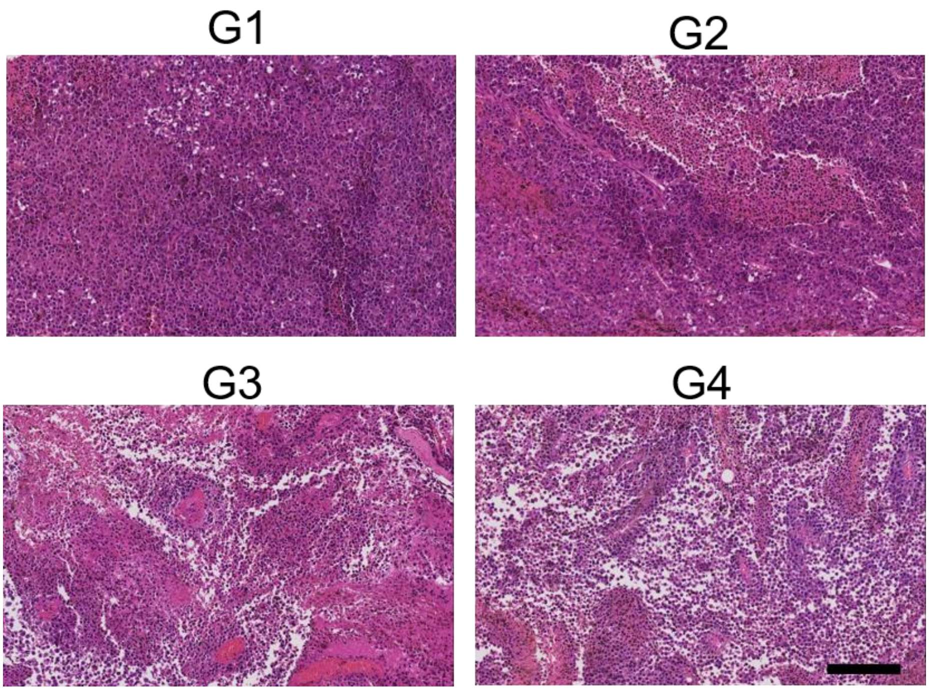

Supplementary Fig. 23 (related to Fig. 5). Representative H&E-stained slice images of tumor tissue after treatment. G1, PBS; G2, HACA-Fe; G3, HACA-GW; G4, HGF. Scale bar: 200 μm. n = 5 biologically independent animals per group.

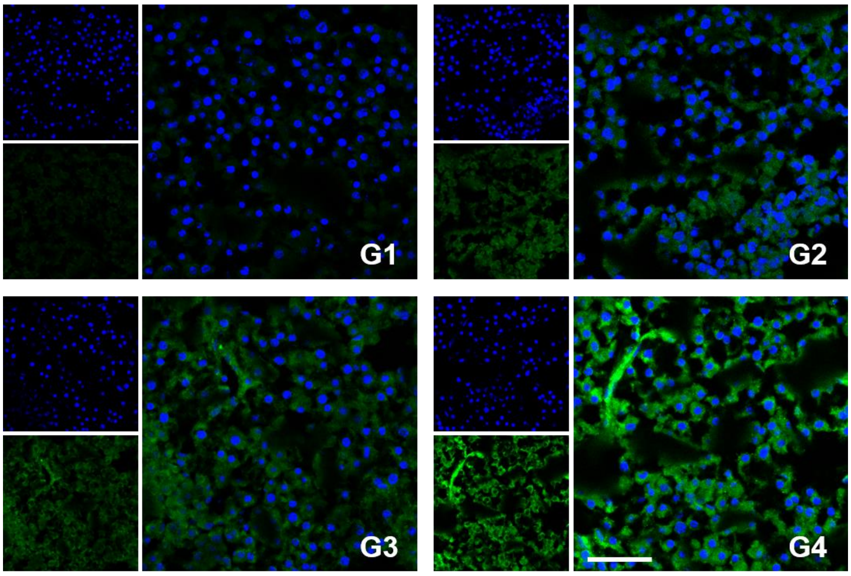

Supplementary Fig. 24 (related to Fig. 5). Representative TUNEL staining of tumor slices obtained from B16F10 tumor-bearing mice after various treatments. G1, PBS; G2, HACA-Fe; G3, HACA-GW; G4, HGF. The scale bar is 50 µm. n = 3 biologically independent animals per group.

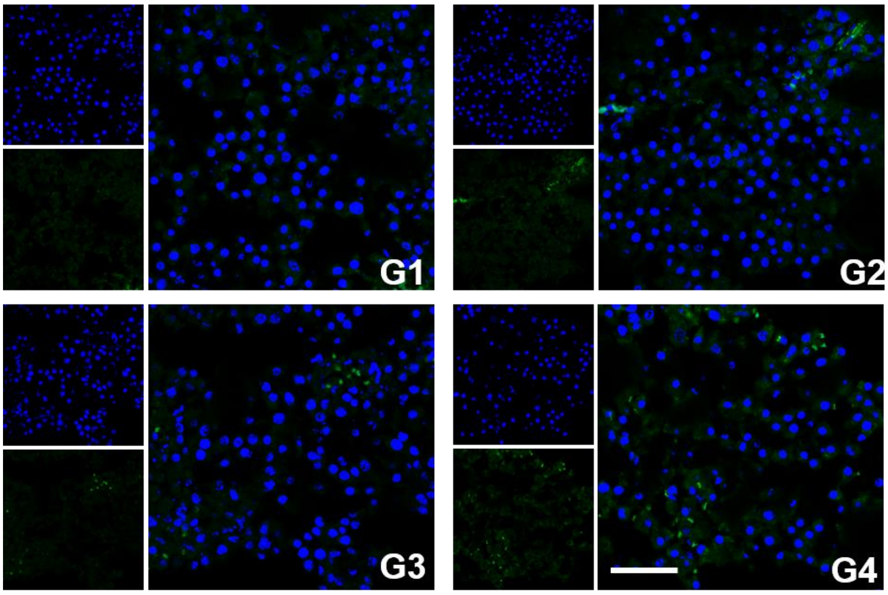

Supplementary Fig. 25 (related to Fig. 5). Cleaved caspase-3 expressions in tumor tissues were detected by immunofluorescence staining after different treatments. G1, PBS; G2, HACA-Fe; G3, HACA-GW; G4, HGF. The scale bar is 50 µm. n = 3 biologically independent animals per group.

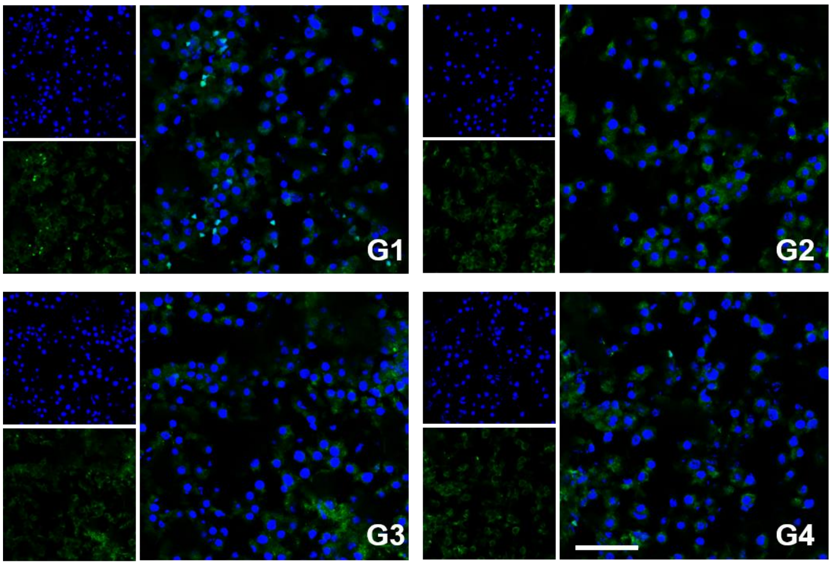

Supplementary Fig. 26 (related to Fig. 5). RIPK3P expressions in tumor tissues were detected by immunofluorescence staining after different treatments. G1, PBS; G2, HACA-Fe; G3, HACA-GW; G4, HGF. n = 3 biologically independent animals per group.

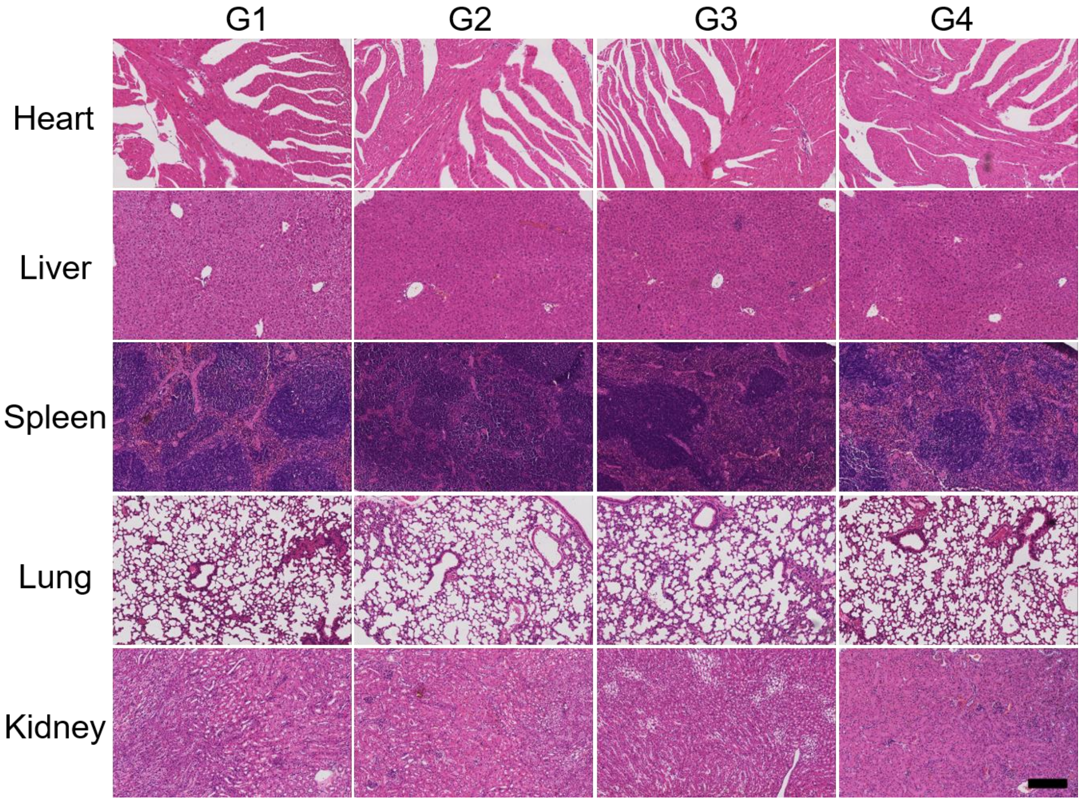

Supplementary Fig. 27 (related to Fig. 5). Representative H&E-stained images of organs harvested from mice at day 14 post-treatment. G1, PBS; G2, HACA-Fe; G3, HACA-GW; G4, HGF. Scale bar: 200 μm. n = 5 biologically independent animals per group.

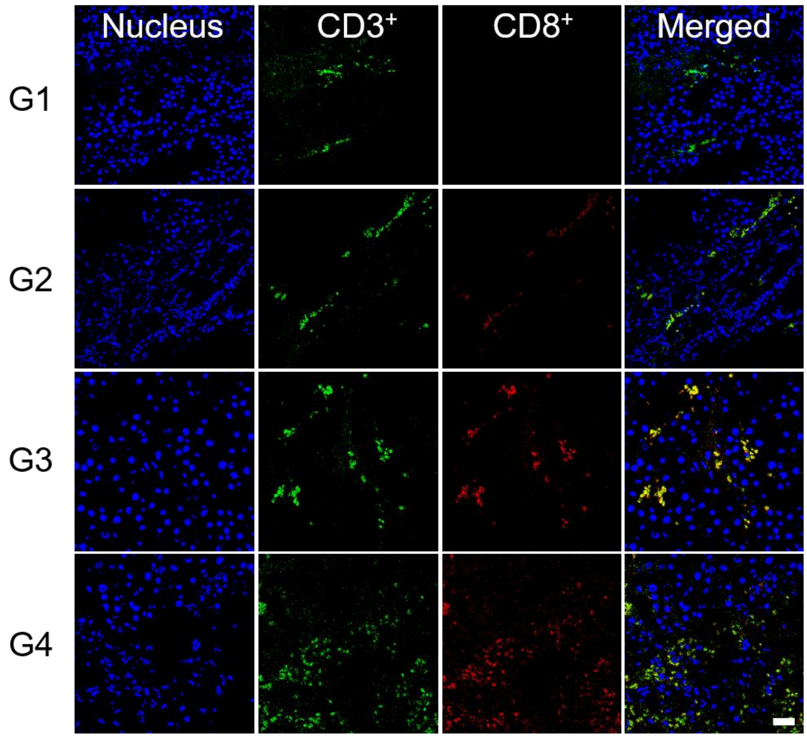

Supplementary Fig. 28 (related to Fig. 5). Representative CLSM images of $CD3^+CD8^+$ T cells in tumor tissues after staining with DAPI (blue), $CD3^+$ antibody (green) and $CD8^+$ antibody (red) respectively. G1, PBS; G2, HACA-Fe; G3, HACA-GW; G4, HGF. The scale bar: 50 μm. n = 3 biologically independent animals per group.

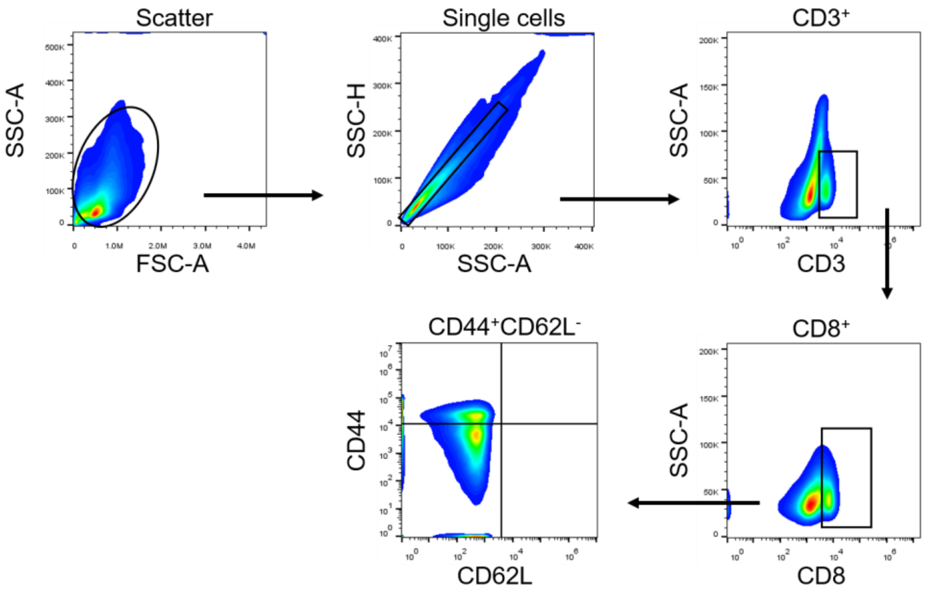

Supplementary Fig. 29 (related to Fig. 5). Representative flow cytometry gating strategies for $CD3^+CD8^+CD44^+CD62L^-$ T cells panel in spleen.

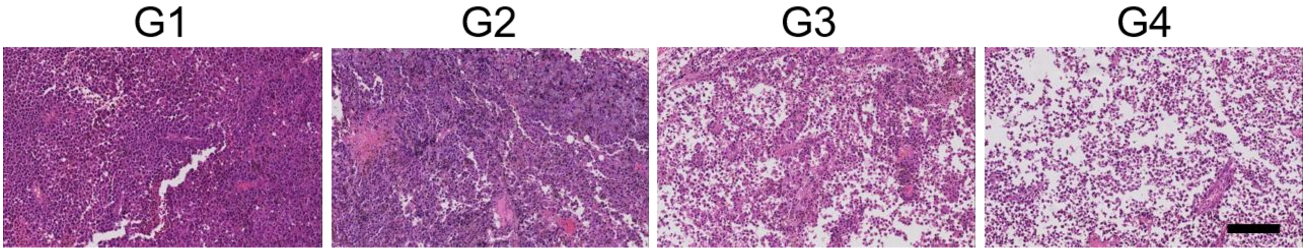

Supplementary Fig. 30 (related to Fig. 6). Representative H&E-stained slice images of tumors after treatment. G1, PBS; G2, anti-PD-L1; G3, HGF; G4, HGF+anti-PD-L1. Scale bar: 200 μm. n = 5 biologically independent animals per group.

.

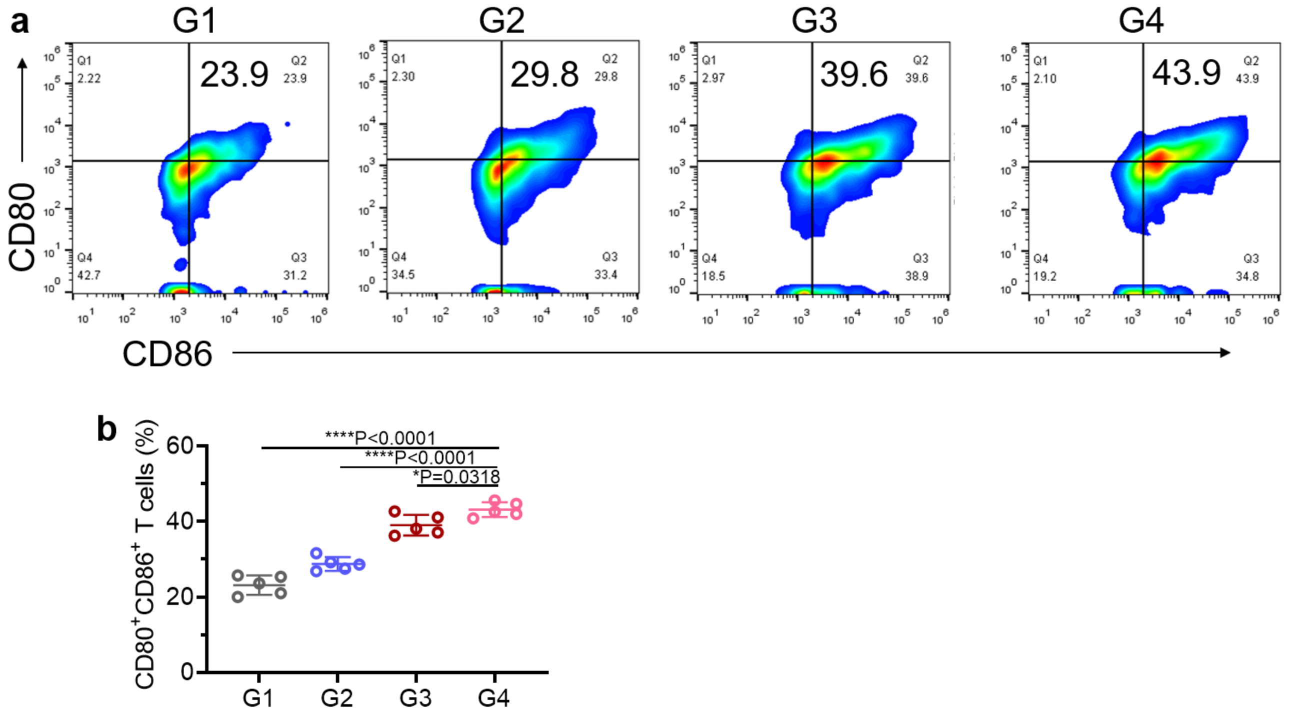

Supplementary Fig. 31 (related to Fig. 6). Representative flow cytometric plots (**a**) and quantification (**b**) of matured DC cells in TDLN after treatment. G1, PBS; G2, anti-PD-L1; G3, HGF; G4, HGF+anti-PD-L1. n = 5 biologically independent animals per group. Data were presented as mean ± SD. Statistically significant differences between groups were identified by one-way ANOVA with Tukey's post hoc test. *P < 0.05, **P < 0.01, ***P < 0.001 and ****P < 0.0001.

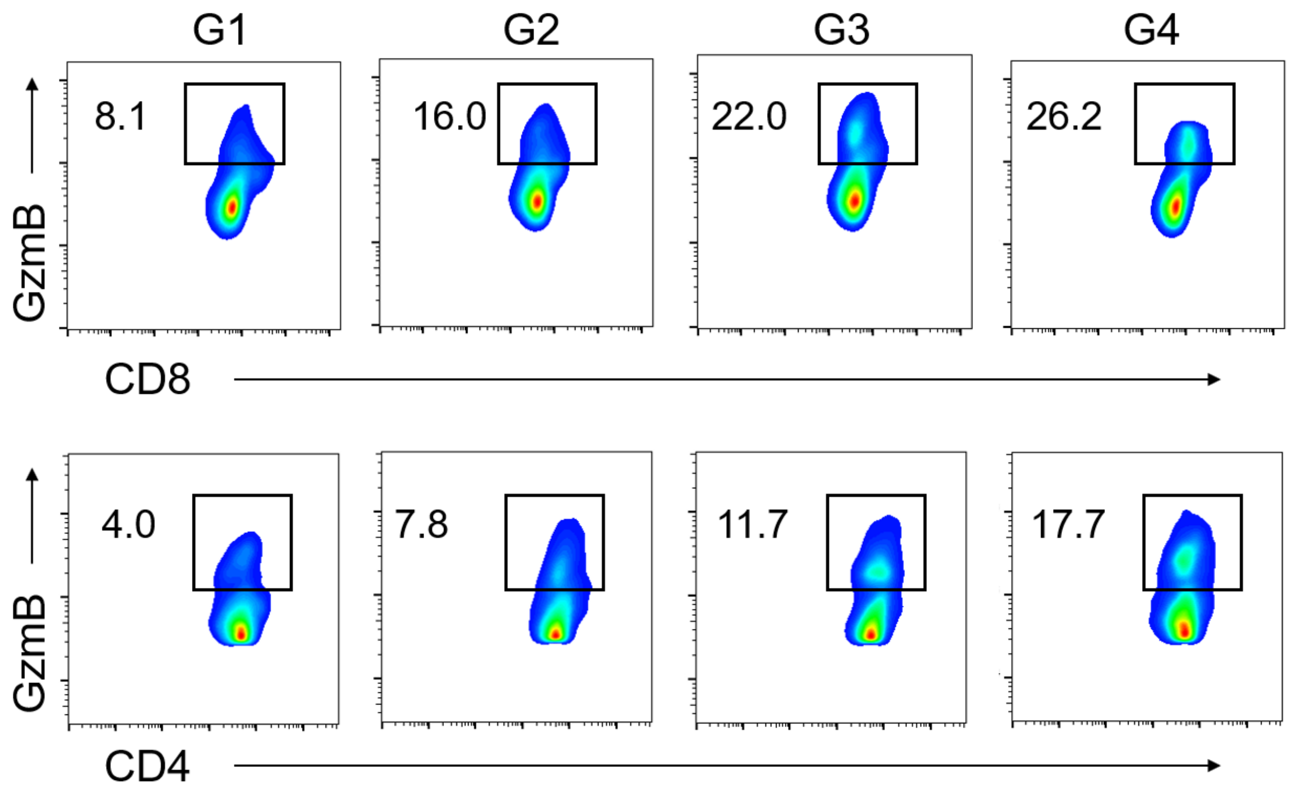

Supplementary Fig. 32 (related to Fig. 6). Representative flow cytometry plots of $CD3^+CD4^+GzmB^+$ and $CD3^+CD8^+GzmB^+$ T cells in TDLN. G1, PBS; G2, anti-PD-L1; G3, HGF; G4, HGF+anti-PD-L1.

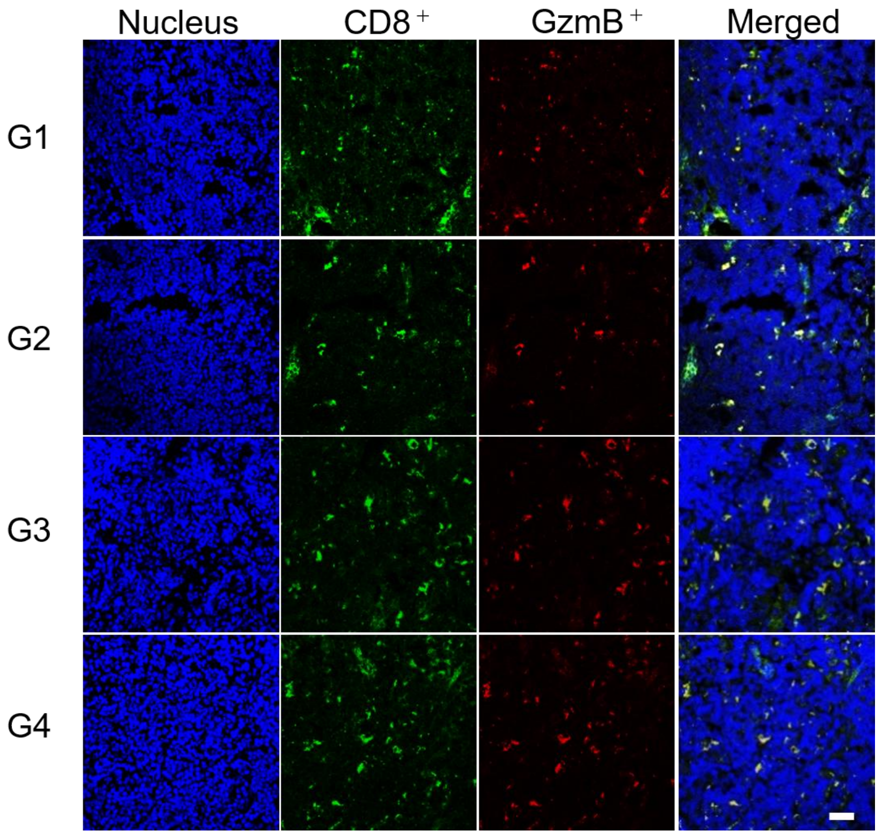

Supplementary Fig. 33 (related to Fig. 6). Representative CLSM images of CD8$^+$GzmB$^+$ T cells after staining with DAPI (blue), CD8$^+$ antibody (green) and GzmB$^+$ antibody (red) respectively. G1, PBS; G2, anti-PD-L1; G3, HGF; G4, HGF+anti-PD-L1. The scale bar: 50 μm. n = 3 biologically independent animals per group.

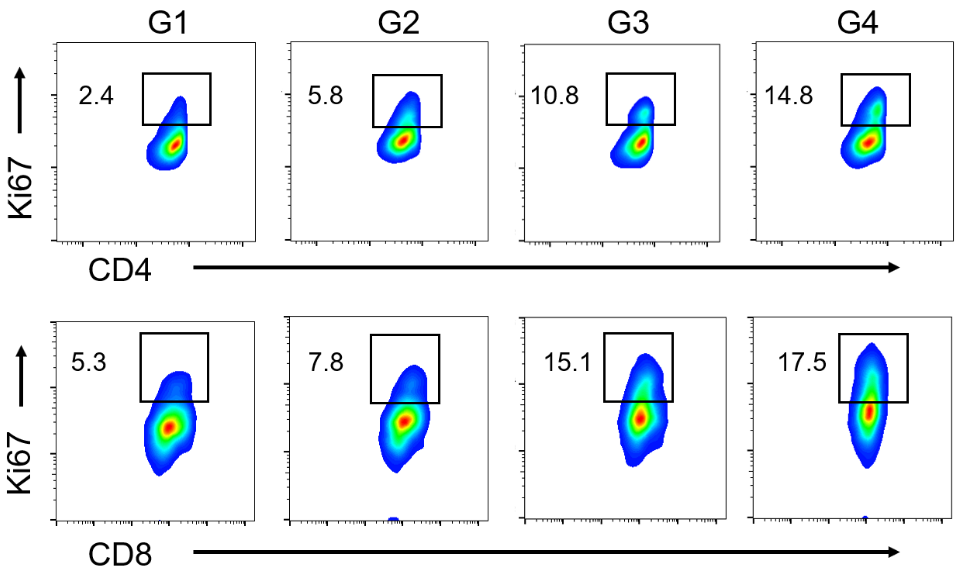

Supplementary Fig. 34 (related to Fig. 6). Representative flow cytometry plots of CD3$^+$CD4$^+$Ki67$^+$ and CD3$^+$CD8$^+$Ki67$^+$ T cells in TDLN. G1, PBS; G2, anti-PD-L1; G3, HGF; G4, HGF+anti-PD-L1.

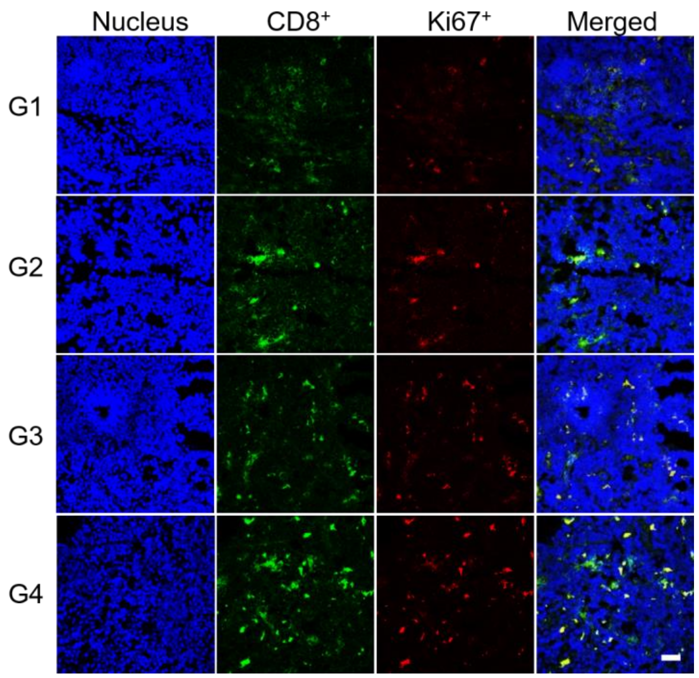

Supplementary Fig. 35 (related to Fig. 6). Representative CLSM images of $CD8^+Ki67^+$ T cells in TDLN after staining with DAPI (blue), $CD8^+$ antibody (green) and $Ki67^+$ antibody (red) respectively. G1, PBS; G2, anti-PD-L1; G3, HGF; G4, HGF+anti-PD-L1. The scale bar: 50 µm. n = 3 biologically independent animals per group.

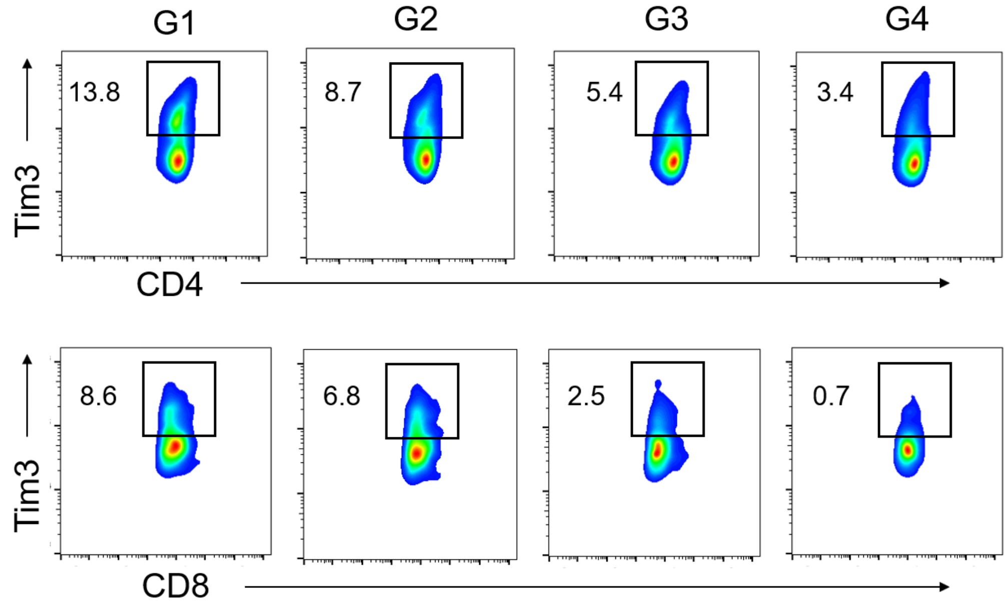

Supplementary Fig. 36 (related to Fig. 6). Representative flow cytometry plots of $CD3^+CD4^+Tim3^+$ and $CD3^+CD8^+Tim3^+$ T cells in TDLN. G1, PBS; G2, anti-PD-L1; G3, HGF; G4, HGF+anti-PD-L1.

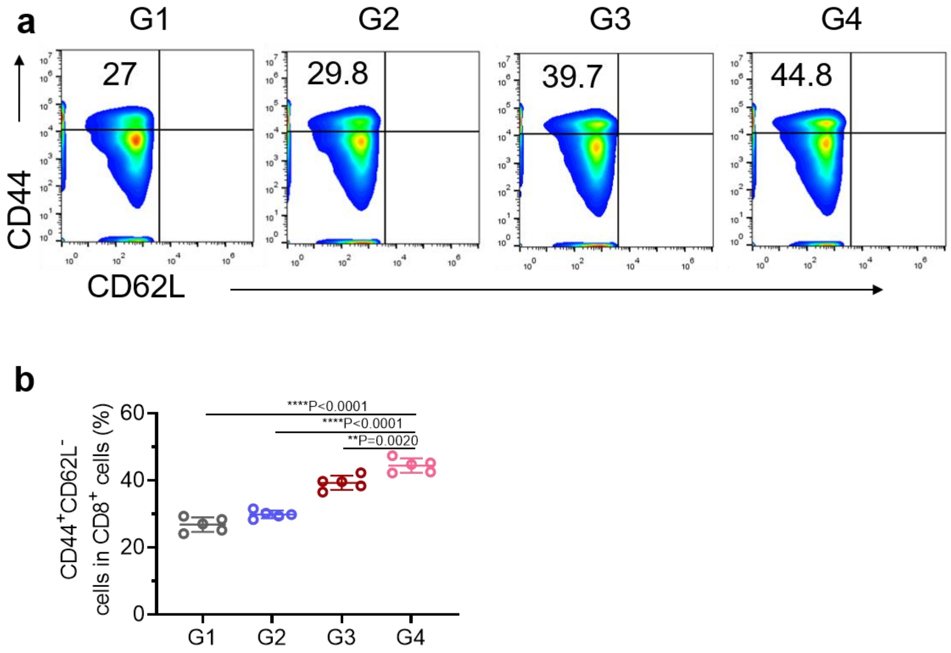

Supplementary Fig. 37 (related to Fig. 6). Representative flow cytometry plots (**a**) and quantification (**b**) of memory T cells ($CD3^+CD8^+CD44^+CD62L^-$, gated on $CD3^+CD8^+$ T cells) in the spleen of mice after treatment. G1, PBS; G2, anti-PD-L1; G3, HGF; G4, HGF+anti-PD-L1. n = 5 biologically independent animals per group. Data were presented as mean ± SD. Statistically significant differences between groups were identified by one-way ANOVA with Tukey's post hoc test. *P < 0.05, **P < 0.01, ***P < 0.001 and ****P < 0.0001.

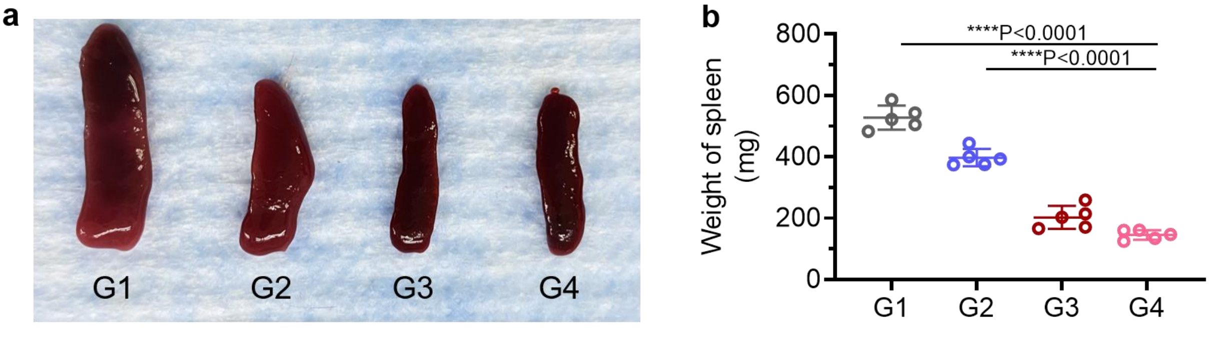

Supplementary Fig. 38 (related to Fig. 6). a Representative photographs of spleens in different groups. G1, PBS; G2, anti-PD-L1; G3, HGF; G4, HGF+anti-PD-L1. n = 5 biologically independent animals per group. **b** The mice spleen weight on day 14 in different treatment groups. n = 5 biologically independent samples per group. Data were presented as mean ± SD. Statistically significant differences between groups were identified by one-way ANOVA with Tukey's post hoc test. *P < 0.05, **P < 0.01, ***P < 0.001 and ****P < 0.0001.

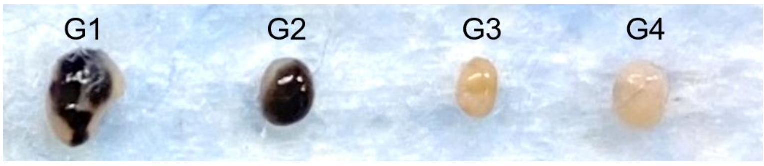

Supplementary Fig. 39 (related to Fig. 6). Representative photographs of TDLNs in different groups. G1, PBS; G2, anti-PD-L1; G3, HGF; G4, HGF+anti-PD-L1. n = 5 biologically independent animals per group.

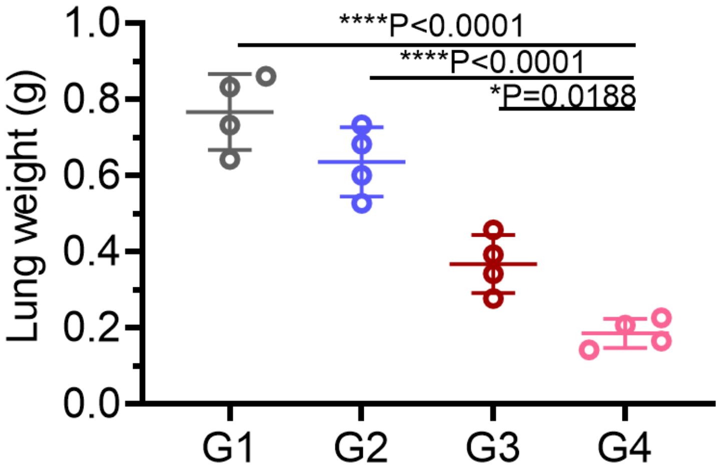

Supplementary Fig. 40 (related to Fig. 7). Lung weight of different groups after treatment on day 21. G1, PBS; G2, anti-PD-L1; G3, HGF; G4, HGF+anti-PD-L1. n = 5 biologically independent animals per group. Data were presented as mean ± SD. Statistically significant differences between groups were identified by one-way ANOVA with Tukey's post hoc test. *P < 0.05, **P < 0.01, ***P < 0.001 and ****P < 0.0001.

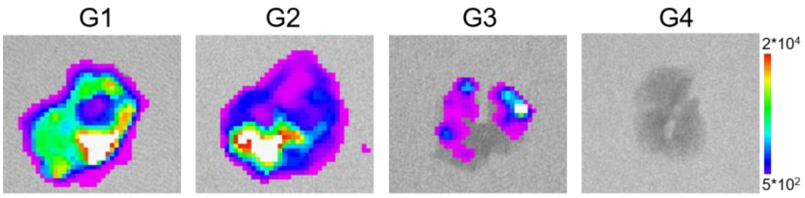

Supplementary Fig. 41 (related to Fig. 7). Respective bioluminescence images of lungs collected from different groups on day 21. G1, PBS; G2, anti-PD-L1; G3, HGF; G4, HGF+anti-PD-L1. n = 4 biologically independent animals per group.
