# Supplementary material for: A nanounit strategy reverses immune suppression of exosomal PD-L1 and is associated with enhanced ferroptosis
Source: Nat Commun. 2021 Sep 30;12:5733. doi: 10.1038/s41467-021-25990-w (PMC8484261; doi:10.1038/s41467-021-25990-w)
Supplement: Supplementary file 3 — Reporting Summary [file 41467_2021_25990_MOESM3_ESM.pdf]

nature research

Corresponding author(s): Yunlu Dai

Last updated by author(s): Sep 13, 2021

# Reporting Summary

Nature Research wishes to improve the reproducibility of the work that we publish. This form provides structure for consistency and transparency in reporting. For further information on Nature Research policies, see our Editorial Policies and the Editorial Policy Checklist.

## Statistics

For all statistical analyses, confirm that the following items are present in the figure legend, table legend, main text, or Methods section.

| n/a | Confirmed | |
|---|---|---|
| ☐ | ☒ | The exact sample size ($n$) for each experimental group/condition, given as a discrete number and unit of measurement |
| ☐ | ☒ | A statement on whether measurements were taken from distinct samples or whether the same sample was measured repeatedly |
| ☐ | ☒ | The statistical test(s) used AND whether they are one- or two-sided<br>*Only common tests should be described solely by name; describe more complex techniques in the Methods section.* |
| ☒ | ☐ | A description of all covariates tested |
| ☐ | ☒ | A description of any assumptions or corrections, such as tests of normality and adjustment for multiple comparisons |
| ☐ | ☒ | A full description of the statistical parameters including central tendency (e.g. means) or other basic estimates (e.g. regression coefficient) AND variation (e.g. standard deviation) or associated estimates of uncertainty (e.g. confidence intervals) |
| ☐ | ☒ | For null hypothesis testing, the test statistic (e.g. $F$, $t$, $r$) with confidence intervals, effect sizes, degrees of freedom and $P$ value noted<br>*Give P values as exact values whenever suitable.* |
| ☒ | ☐ | For Bayesian analysis, information on the choice of priors and Markov chain Monte Carlo settings |
| ☒ | ☐ | For hierarchical and complex designs, identification of the appropriate level for tests and full reporting of outcomes |
| ☒ | ☐ | Estimates of effect sizes (e.g. Cohen's $d$, Pearson's $r$), indicating how they were calculated |

*Our web collection on statistics for biologists contains articles on many of the points above.*

## Software and code

Policy information about availability of computer code

| | |
|---|---|
| Data collection | Bruker In-Vivo Xtreme System; Zen software. |
| Data analysis | All statistical analyses were performed on Graphpad Prism (v8.0). All flow cytometry data were analyzed on FlowJo software (v10.0; BD Biosciences, USA). Bruker In-Vivo Xtreme System was used to analyze fluorescent and bioluminescent images. |

For manuscripts utilizing custom algorithms or software that are central to the research but not yet described in published literature, software must be made available to editors and reviewers. We strongly encourage code deposition in a community repository (e.g. GitHub). See the Nature Research guidelines for submitting code & software for further information.

## Data

Policy information about availability of data

All manuscripts must include a data availability statement. This statement should provide the following information, where applicable:

- Accession codes, unique identifiers, or web links for publicly available datasets
- A list of figures that have associated raw data
- A description of any restrictions on data availability

The pertinent data supporting the findings of this study are included in the article and the supplementary information files. All data generated in this study are provided as a Source data file. Source data are provided with this paper and available from figshare with the identifier https://figshare.com/s/609c457b4a3698a2b086.



April 2020

# Field-specific reporting

Please select the one below that is the best fit for your research. If you are not sure, read the appropriate sections before making your selection.

- [x] Life sciences
- [ ] Behavioural & social sciences
- [ ] Ecological, evolutionary & environmental sciences

For a reference copy of the document with all sections, see nature.com/documents/nr-reporting-summary-flat.pdf

# Life sciences study design

All studies must disclose on these points even when the disclosure is negative.

| | |
|---|---|
| Sample size | No statistical methods were used to pre-determine sample sizes and the sample sizes in the study were chosen based on experience from previous in house studies and to balance between the ability to detect significance differences while reducing the number of animals used. |
| Data exclusions | No data were excluded. |
| Replication | Experiments were repeated at least three times, unless otherwise stated in the respective figure legend. Replicates were reproducible. |
| Randomization | Cells and mice were allocated randomly into each treatment groups. |
| Blinding | For most parts, investigators were not blinded during data collection or analysis because planning, execution, and analysis of the studies was performed by the same personnel. Tumor volume measurements were performed by independent researchers who were blinded as to treatment group assignment. Independent researchers who were unaware of the treatment conditions conducted the bioluminescence imaging. |

# Behavioural & social sciences study design

All studies must disclose on these points even when the disclosure is negative.

| | |
|---|---|
| Study description | *Briefly describe the study type including whether data are quantitative, qualitative, or mixed-methods (e.g. qualitative cross-sectional, quantitative experimental, mixed-methods case study).* |
| Research sample | *State the research sample (e.g. Harvard university undergraduates, villagers in rural India) and provide relevant demographic information (e.g. age, sex) and indicate whether the sample is representative. Provide a rationale for the study sample chosen. For studies involving existing datasets, please describe the dataset and source.* |
| Sampling strategy | *Describe the sampling procedure (e.g. random, snowball, stratified, convenience). Describe the statistical methods that were used to predetermine sample size OR if no sample-size calculation was performed, describe how sample sizes were chosen and provide a rationale for why these sample sizes are sufficient. For qualitative data, please indicate whether data saturation was considered, and what criteria were used to decide that no further sampling was needed.* |
| Data collection | *Provide details about the data collection procedure, including the instruments or devices used to record the data (e.g. pen and paper, computer, eye tracker, video or audio equipment) whether anyone was present besides the participant(s) and the researcher, and whether the researcher was blind to experimental condition and/or the study hypothesis during data collection.* |
| Timing | *Indicate the start and stop dates of data collection. If there is a gap between collection periods, state the dates for each sample cohort.* |
| Data exclusions | *If no data were excluded from the analyses, state so OR if data were excluded, provide the exact number of exclusions and the rationale behind them, indicating whether exclusion criteria were pre-established.* |
| Non-participation | *State how many participants dropped out/declined participation and the reason(s) given OR provide response rate OR state that no participants dropped out/declined participation.* |
| Randomization | *If participants were not allocated into experimental groups, state so OR describe how participants were allocated to groups, and if allocation was not random, describe how covariates were controlled.* |

# Ecological, evolutionary & environmental sciences study design

All studies must disclose on these points even when the disclosure is negative.

| | |
|---|---|
| Study description | *Briefly describe the study. For quantitative data include treatment factors and interactions, design structure (e.g. factorial, nested, hierarchical), nature and number of experimental units and replicates.* |

| | |
|---|---|
| Research sample | *Describe the research sample (e.g. a group of tagged Passer domesticus, all Stenocereus thurberi within Organ Pipe Cactus National Monument), and provide a rationale for the sample choice. When relevant, describe the organism taxa, source, sex, age range and any manipulations. State what population the sample is meant to represent when applicable. For studies involving existing datasets, describe the data and its source.* |
| Sampling strategy | *Note the sampling procedure. Describe the statistical methods that were used to predetermine sample size OR if no sample-size calculation was performed, describe how sample sizes were chosen and provide a rationale for why these sample sizes are sufficient.* |
| Data collection | *Describe the data collection procedure, including who recorded the data and how.* |
| Timing and spatial scale | *Indicate the start and stop dates of data collection, noting the frequency and periodicity of sampling and providing a rationale for these choices. If there is a gap between collection periods, state the dates for each sample cohort. Specify the spatial scale from which the data are taken* |
| Data exclusions | *If no data were excluded from the analyses, state so OR if data were excluded, describe the exclusions and the rationale behind them, indicating whether exclusion criteria were pre-established.* |
| Reproducibility | *Describe the measures taken to verify the reproducibility of experimental findings. For each experiment, note whether any attempts to repeat the experiment failed OR state that all attempts to repeat the experiment were successful.* |
| Randomization | *Describe how samples/organisms/participants were allocated into groups. If allocation was not random, describe how covariates were controlled. If this is not relevant to your study, explain why.* |
| Blinding | *Describe the extent of blinding used during data acquisition and analysis. If blinding was not possible, describe why OR explain why blinding was not relevant to your study.* |

Did the study involve field work? ☐ Yes ☐ No

## Field work, collection and transport

| | |
|---|---|
| Field conditions | *Describe the study conditions for field work, providing relevant parameters (e.g. temperature, rainfall).* |
| Location | *State the location of the sampling or experiment, providing relevant parameters (e.g. latitude and longitude, elevation, water depth).* |
| Access & import/export | *Describe the efforts you have made to access habitats and to collect and import/export your samples in a responsible manner and in compliance with local, national and international laws, noting any permits that were obtained (give the name of the issuing authority, the date of issue, and any identifying information).* |
| Disturbance | *Describe any disturbance caused by the study and how it was minimized.* |

# Reporting for specific materials, systems and methods

We require information from authors about some types of materials, experimental systems and methods used in many studies. Here, indicate whether each material, system or method listed is relevant to your study. If you are not sure if a list item applies to your research, read the appropriate section before selecting a response.

### Materials & experimental systems

| n/a | Involved in the study | |
|---|---|---|
| ☐ | ☒ | Antibodies |
| ☐ | ☒ | Eukaryotic cell lines |
| ☒ | ☐ | Palaeontology and archaeology |
| ☐ | ☒ | Animals and other organisms |
| ☒ | ☐ | Human research participants |
| ☒ | ☐ | Clinical data |
| ☒ | ☐ | Dual use research of concern |

### Methods

| n/a | Involved in the study | |
|---|---|---|
| ☒ | ☐ | ChIP-seq |
| ☐ | ☒ | Flow cytometry |
| ☒ | ☐ | MRI-based neuroimaging |

## Antibodies

| | |
|---|---|
| Antibodies used | The following primary antibodies were used for flow cytometry: CD45 (Biolegend, cat. no. 103101); CD11c (Biolegend, N418, cat. no. 117306); CD80 (Biolegend, 16–10A1, cat. no. 104705); CD86 (Biolegend, GL-1, cat. no. 105005); CD4 (eBioscience, cat. no. 11-0041-82); CD8 (eBioscience, cat. no. 45-0081-82); IFN-γ (R&D Systems, cat. no. IC485P-100); Tim-3 (Biolegend, cat. no. 119718); Ki-67 (Biolegend, cat. no. 652411); Granzyme B (Biolegend, cat. no. 652411); CD3 (Biolegend, cat. no. 100307); CD44 (eBioscience, cat. no. 12-0441-81); CD62L-APC (eBioscience, cat. no. 17-0621-81).<br>The following primary antibodies were used for western blot: CD63 (Biolegend, cat. no. 143902); PD-L1 (Clone no. EPR20529, abcam, cat. no. ab213480); GAPDH (Santa Cruz Biotechnology, cat. no. sc-365062).<br>The HRP conjugated secondary antibodies were used for western blot: HRP Goat anti-rat IgG Antibody (Biolegend, cat. no. 405405); HRP Donkey anti-rabbit IgG Antibody (Biolegend, cat. no. 406401); HRP Goat anti-mouse IgG Antibody (Biolegend, cat. no. 405306). |

The following primary antibodies were used for ELISA: TNF-α Mouse ELISA Kit (Invitrogen, cat. no. BMS607-3); IL-6 Mouse ELISA Kit (Invitrogen, cat. no. BMS603-2); IL-12 Mouse ELISA Kit (Invitrogen, cat. no. BMS616); IFN gamma Mouse ELISA Kit (Invitrogen, cat. no. BMS606).
The following antibodies were used for stimulation: CD3 (eBioscience, cat. no. 11-0031-82); CD28 (eBioscience, cat. no. 16-0281-82)
The following primary antibody was used for immune checkpoint blockade therapy: PD-L1 (Clone. 10F.9G2, leinco, cat. no. P363).

Validation

All antibodies are from commercial sources and their validation data are available on the manufacturer's website.
CD45 (Rat, FC/IHC-F/CyTOF/CMCD/IHC/WB, 1/50, Biolegend, cat. no. 103101): https://www.biolegend.com/en-us/products/purified-anti-mouse-cd45-antibody-102?GroupID=BLG1932
CD11c (Armenian Hamster, FC, Biolegend, 1/50, cat. no. 117306): https://www.biolegend.com/en-us/search-results/fitc-anti-mouse-cd11c-antibody-1815?GroupID=BLG11937
CD80 (Armenian Hamster, FC, Biolegend, 16–10A1, 1/100, cat. no. 104705): https://www.biolegend.com/en-us/search-results/fitc-anti-mouse-cd80-antibody-41
CD86 (Rat, FC, Biolegend, GL-1, 1/100, cat. no. 105005): https://www.biolegend.com/en-us/products/fitc-anti-mouse-cd86-antibody-254?GroupID=BLG11928
CD4 (Rat, IHC/IHC(P)/IHC(F)/ICC/IF/Flow/FN/Misc, 1/50, eBioscience, cat. no. 11-0041-82): https://www.thermofisher.com/antibody/product/CD4-Antibody-clone-GK1-5-Monoclonal/11-0041-82
CD8 (Rat, IHC/ICC/IF/Flow/FN/Misc, eBioscience, 1/50, cat. no. 45-0081-82): https://www.thermofisher.com/antibody/product/CD8a-Antibody-clone-53-6-7-Monoclonal/45-0081-82
IFN-γ (Rat, FC, R&D Systems, 1/50, cat. no. IC485P-100): https://www.rndsystems.com/products/mouse-ifn-gamma-pe-conjugated-antibody-37895_ic485p
Tim-3 (Rat, FC, Biolegend, 1/100, cat. no. 119718): https://www.biolegend.com/en-us/products/percp-cyanine5-5-anti-mouse-cd366-tim-3-antibody-13043?GroupID=BLG10787
Ki-67 (Rat, ICFC, Biolegend, 1/100, cat. no. 652411): https://www.biolegend.com/en-us/products/brilliant-violet-421-anti-mouse-ki-67-antibody-8982?GroupID=GROUP26
Granzyme B (Mouse, ICFC, 1/50, Biolegend, cat. no. 652411): https://www.biolegend.com/en-us/products/fitc-anti-human-mouse-granzyme-b-antibody-6066?GroupID=BLG15670
CD3 (Armenian Hamster, FC, 1/50, Biolegend, cat. no. 100307): https://www.biolegend.com/en-us/products/pe-anti-mouse-cd3epsilon-antibody-25?GroupID=BLG6744
CD44 (Rat, ICC/IF/Flow/Misc, 1/100, eBioscience, cat. no. 12-0441-81): https://www.thermofisher.com/antibody/product/CD44-Antibody-clone-IM7-Monoclonal/12-0441-82
CD62L-APC (Rat, ICC/IF/Flow, 1/50, eBioscience, cat. no. 17-0621-81): https://www.thermofisher.com/order/genome-database/dataSheetPdf?producttype=antibody&productsubtype=antibody_primary&productId=17-0621-81&version=65
CD63 (Rat, ICFC/FC/IHC-F/WB, 1/2000, Biolegend, cat. no. 143902): https://www.biolegend.com/en-us/search-results/purified-anti-mouse-cd63-antibody-7813
PD-L1 (Rabbit, ICC/IF/IP/WB, 1/1000, abcam, cat. no. ab213480): https://www.abcam.com/pd-l1-antibody-epr20529-ab213480.html
GAPDH (mouse, WB/IP/IF/IHC(P)/ELISA, 1/1000, Santa Cruz Biotechnology, cat no. sc-365062): https://www.scbt.com/p/gapdh-antibody-g-9?requestFrom=search
HRP Goat anti-rat IgG Antibody (Goat, WB/IHC/ELISA, 1/2000, Biolegend, cat. no. 405405): https://www.biolegend.com/en-us/products/hrp-goat-anti-rat-igg-minimal-x-reactivity-1396
HRP Donkey anti-rabbit IgG Antibody (Donkey, WB/IHC/ELISA, 1/2000, Biolegend, cat. no. 406401): https://www.biolegend.com/en-us/products/hrp-donkey-anti-rabbit-igg-minimal-x-reactivity-2264
HRP Goat anti-mouse IgG Antibody (Goat, WB/IHC/ELISA, 1/2000, Biolegend, cat. no. 405306): https://www.biolegend.com/en-us/products/hrp-goat-anti-mouse-igg-minimal-x-reactivity-1395
TNF-α Mouse ELISA Kit (Invitrogen, cat. no. BMS607-3): https://www.thermofisher.com/elisa/product/TNF-alpha-Mouse-ELISA-Kit/BMS607-3
IL-6 Mouse ELISA Kit (Invitrogen, cat. no. BMS603-2): https://www.thermofisher.com/elisa/product/IL-6-Mouse-ELISA-Kit/BMS603-2
IL-12 Mouse ELISA Kit (Invitrogen, cat. no. BMS616): https://www.thermofisher.com/elisa/product/IL-12-p40-70-IL-12B-Mouse-ELISA-Kit/EMIL12B
IFN gamma Mouse ELISA Kit (Invitrogen, cat. no. BMS606). https://www.thermofisher.com/elisa/product/IFN-gamma-Mouse-ELISA-Kit/BMS606
CD3 (Armenian Hamster, Flow/IP/FN/TCA/Misc/ICC/IF/IHC/WB, eBioscience, cat. no. 11-0031-82): https://www.thermofisher.com/antibody/product/CD3e-Antibody-clone-145-2C11-Monoclonal/11-0031-82
CD28 (Syrian hamster, WB/ Flow/Elisa/FN/IV/Misc eBioscience, cat. no. 16-0281-82): https://www.thermofisher.com/antibody/product/CD28-Antibody-clone-37-51-Monoclonal/16-0281-82
PD-L1 (Rat, B/CODEX/FC/IHC/FF/WB, leinco, cat. no. P363): https://www.leinco.com/p/anti-mouse-pd-l1-cd274-purified-functional-grade-gold/

## Eukaryotic cell lines

Policy information about cell lines

| | |
|---|---|
| Cell line source(s) | B16F10 cell lines were purchased from ATCC. B16F10-fLuc cell lines were obtained from Dr. Lisi Xie at University of Macau. |
| Authentication | STR testing. |
| Mycoplasma contamination | All cell lines in this study get tested without mycoplasma contamination. |
| Commonly misidentified lines (See ICLAC register) | No commonly misidentified cell lines were used. |

## Palaeontology and Archaeology

| | |
|---|---|
| Specimen provenance | *Provide provenance information for specimens and describe permits that were obtained for the work (including the name of the issuing authority, the date of issue, and any identifying information).* |
| Specimen deposition | *Indicate where the specimens have been deposited to permit free access by other researchers.* |
| Dating methods | *If new dates are provided, describe how they were obtained (e.g. collection, storage, sample pretreatment and measurement), where they were obtained (i.e. lab name), the calibration program and the protocol for quality assurance OR state that no new dates are provided.* |

☐ Tick this box to confirm that the raw and calibrated dates are available in the paper or in Supplementary Information.

| | |
|---|---|
| Ethics oversight | *Identify the organization(s) that approved or provided guidance on the study protocol, OR state that no ethical approval or guidance was required and explain why not.* |

Note that full information on the approval of the study protocol must also be provided in the manuscript.

## Animals and other organisms

Policy information about studies involving animals; ARRIVE guidelines recommended for reporting animal research

| | |
|---|---|
| Laboratory animals | Female C57BL/6J mice (6–8 weeks) were supplied by the Animal Center in the Faculty of Health Sciences, University of Macau. Mice were housed in a Specific-pathogen-free (SPF) facility at 23-25°C on a 12-h light/dark cycle with unrestricted access to food and water. Temperature in animal facilities was 20-24 °C and humidity was 55 ± 10%. |
| Wild animals | No wild animal was involved in this study. |
| Field-collected samples | No field-collected sample was included in this study. |
| Ethics oversight | Animal experimental procedures were performed via following a protocol (UMARE-030-2018) approved by the Animal Ethics Committee of University of Macau. |

Note that full information on the approval of the study protocol must also be provided in the manuscript.

## Human research participants

Policy information about studies involving human research participants

| | |
|---|---|
| Population characteristics | *Describe the covariate-relevant population characteristics of the human research participants (e.g. age, gender, genotypic information, past and current diagnosis and treatment categories). If you filled out the behavioural & social sciences study design questions and have nothing to add here, write "See above."* |
| Recruitment | *Describe how participants were recruited. Outline any potential self-selection bias or other biases that may be present and how these are likely to impact results.* |
| Ethics oversight | *Identify the organization(s) that approved the study protocol.* |

Note that full information on the approval of the study protocol must also be provided in the manuscript.

## Clinical data

Policy information about clinical studies

All manuscripts should comply with the ICMJEguidelines for publication of clinical research and a completedCONSORT checklist must be included with all submissions.

| | |
|---|---|
| Clinical trial registration | *Provide the trial registration number from ClinicalTrials.gov or an equivalent agency.* |
| Study protocol | *Note where the full trial protocol can be accessed OR if not available, explain why.* |
| Data collection | *Describe the settings and locales of data collection, noting the time periods of recruitment and data collection.* |
| Outcomes | *Describe how you pre-defined primary and secondary outcome measures and how you assessed these measures.* |

## Dual use research of concern

Policy information about dual use research of concern

### Hazards

Could the accidental, deliberate or reckless misuse of agents or technologies generated in the work, or the application of information presented in the manuscript, pose a threat to:

| No | Yes | |
|---|---|---|
| ☐ | ☐ | Public health |
| ☐ | ☐ | National security |
| ☐ | ☐ | Crops and/or livestock |
| ☐ | ☐ | Ecosystems |
| ☐ | ☐ | Any other significant area |

## Experiments of concern

Does the work involve any of these experiments of concern:

| No | Yes | |
|---|---|---|
| ☐ | ☐ | Demonstrate how to render a vaccine ineffective |
| ☐ | ☐ | Confer resistance to therapeutically useful antibiotics or antiviral agents |
| ☐ | ☐ | Enhance the virulence of a pathogen or render a nonpathogen virulent |
| ☐ | ☐ | Increase transmissibility of a pathogen |
| ☐ | ☐ | Alter the host range of a pathogen |
| ☐ | ☐ | Enable evasion of diagnostic/detection modalities |
| ☐ | ☐ | Enable the weaponization of a biological agent or toxin |
| ☐ | ☐ | Any other potentially harmful combination of experiments and agents |

# ChIP-seq

## Data deposition

☐ Confirm that both raw and final processed data have been deposited in a public database such as GEO.

☐ Confirm that you have deposited or provided access to graph files (e.g. BED files) for the called peaks.

| | |
|---|---|
| Data access links<br>*May remain private before publication.* | *For "Initial submission" or "Revised version" documents, provide reviewer access links. For your "Final submission" document, provide a link to the deposited data.* |
| Files in database submission | *Provide a list of all files available in the database submission.* |
| Genome browser session<br>(e.g. UCSC) | *Provide a link to an anonymized genome browser session for "Initial submission" and "Revised version" documents only, to enable peer review. Write "no longer applicable" for "Final submission" documents.* |

## Methodology

| | |
|---|---|
| Replicates | *Describe the experimental replicates, specifying number, type and replicate agreement.* |
| Sequencing depth | *Describe the sequencing depth for each experiment, providing the total number of reads, uniquely mapped reads, length of reads and whether they were paired- or single-end.* |
| Antibodies | *Describe the antibodies used for the ChIP-seq experiments; as applicable, provide supplier name, catalog number, clone name, and lot number.* |
| Peak calling parameters | *Specify the command line program and parameters used for read mapping and peak calling, including the ChIP, control and index files used.* |
| Data quality | *Describe the methods used to ensure data quality in full detail, including how many peaks are at FDR 5% and above 5-fold enrichment.* |
| Software | *Describe the software used to collect and analyze the ChIP-seq data. For custom code that has been deposited into a community repository, provide accession details.* |

# Flow Cytometry

## Plots

Confirm that:

- [x] The axis labels state the marker and fluorochrome used (e.g. CD4-FITC).
- [x] The axis scales are clearly visible. Include numbers along axes only for bottom left plot of group (a 'group' is an analysis of identical markers).
- [x] All plots are contour plots with outliers or pseudocolor plots.
- [x] A numerical value for number of cells or percentage (with statistics) is provided.

## Methodology

| | |
|---|---|
| Sample preparation | For tissue samples, the tissue were collected and incubated in dissociation buffer with 1640 medium (contained collagenase (1 mg ml-1), hyaluronidase (100 U), and deoxyribonuclease I (100 µg ml-1)) at 37 °C for digesting tissue. The cell suspension was mechanically minced against a 70 µm cell strainer to obtain single cell suspension and treated with ACK lysis buffer (Gibco). The single cell suspension was washed with FACS buffer and then stained with indicated antibodies following the manufacturer's instructions. |
| Instrument | BD Accuri C6 Plus Flow cytometer (BD Biosciences) |
| Software | FlowJo (v10; BD Biosciences, USA) |
| Cell population abundance | No sorting was performed. |
| Gating strategy | Cells was first gated on FSC-A and SSC-A. Single cells were usually gated using SSC-H and SSC-A. |

- [x] Tick this box to confirm that a figure exemplifying the gating strategy is provided in the Supplementary Information.

# Magnetic resonance imaging

## Experimental design

| | |
|---|---|
| Design type | *Indicate task or resting state; event-related or block design.* |
| Design specifications | *Specify the number of blocks, trials or experimental units per session and/or subject, and specify the length of each trial or block (if trials are blocked) and interval between trials.* |
| Behavioral performance measures | *State number and/or type of variables recorded (e.g. correct button press, response time) and what statistics were used to establish that the subjects were performing the task as expected (e.g. mean, range, and/or standard deviation across subjects).* |

## Acquisition

| | |
|---|---|
| Imaging type(s) | *Specify: functional, structural, diffusion, perfusion.* |
| Field strength | *Specify in Tesla* |
| Sequence & imaging parameters | *Specify the pulse sequence type (gradient echo, spin echo, etc.), imaging type (EPI, spiral, etc.), field of view, matrix size, slice thickness, orientation and TE/TR/flip angle.* |
| Area of acquisition | *State whether a whole brain scan was used OR define the area of acquisition, describing how the region was determined.* |

Diffusion MRI  [ ] Used  [ ] Not used

## Preprocessing

| | |
|---|---|
| Preprocessing software | *Provide detail on software version and revision number and on specific parameters (model/functions, brain extraction, segmentation, smoothing kernel size, etc.).* |
| Normalization | *If data were normalized/standardized, describe the approach(es): specify linear or non-linear and define image types used for transformation OR indicate that data were not normalized and explain rationale for lack of normalization.* |
| Normalization template | *Describe the template used for normalization/transformation, specifying subject space or group standardized space (e.g. original Talairach, MNI305, ICBM152) OR indicate that the data were not normalized.* |
| Noise and artifact removal | *Describe your procedure(s) for artifact and structured noise removal, specifying motion parameters, tissue signals and physiological signals (heart rate, respiration).* |

Volume censoring | *Define your software and/or method and criteria for volume censoring, and state the extent of such censoring.*

## Statistical modeling & inference

Model type and settings | *Specify type (mass univariate, multivariate, RSA, predictive, etc.) and describe essential details of the model at the first and second levels (e.g. fixed, random or mixed effects; drift or auto-correlation).*

Effect(s) tested | *Define precise effect in terms of the task or stimulus conditions instead of psychological concepts and indicate whether ANOVA or factorial designs were used.*

Specify type of analysis: ☐ Whole brain ☐ ROI-based ☐ Both

Statistic type for inference
(See Eklund et al. 2016) | *Specify voxel-wise or cluster-wise and report all relevant parameters for cluster-wise methods.*

Correction | *Describe the type of correction and how it is obtained for multiple comparisons (e.g. FWE, FDR, permutation or Monte Carlo).*

## Models & analysis

| n/a | Involved in the study |
|---|---|
| ☐ | ☐ Functional and/or effective connectivity |
| ☐ | ☐ Graph analysis |
| ☐ | ☐ Multivariate modeling or predictive analysis |

Functional and/or effective connectivity | *Report the measures of dependence used and the model details (e.g. Pearson correlation, partial correlation, mutual information).*

Graph analysis | *Report the dependent variable and connectivity measure, specifying weighted graph or binarized graph, subject- or group-level, and the global and/or node summaries used (e.g. clustering coefficient, efficiency, etc.).*

Multivariate modeling and predictive analysis | *Specify independent variables, features extraction and dimension reduction, model, training and evaluation metrics.*
